# Eliminating the Imbalanced Mobility Bottlenecks via Reshaping Internal Potential Distribution in Organic Photovoltaics

**DOI:** 10.1002/advs.202302880

**Published:** 2023-08-27

**Authors:** Yu Cui, Chao Zhao, João Paulo Araújo Souza, Leandro Benatto, Marlus Koehler, Wei Ma, Han Yan

**Affiliations:** ^1^ State Key Laboratory for Mechanical Behavior of Materials School of Materials Science and Engineering Xi'an Jiaotong University Xi'an 710049 P. R. China; ^2^ Department of Physics Federal University of Paraná Curitiba 81531‐980 Brazil

**Keywords:** dilute bulk heterojunction, electrical doping, imbalanced carrier mobility, internal potential distribution, organic solar cell, semitransparent device

## Abstract

The imbalanced carrier mobility remains a bottleneck for performance breakthrough in even those organic solar cells (OSCs) with recorded power conversion efficiencies (PCEs). Herein, a counter electrode doping strategy is proposed to reshape the internal potential distribution, which targets to extract the low mobility carriers at far end. Device simulations reveal that the key of this strategy is to partially dope the active layer with a certain depth, therefore it strengthens the electric field for low mobility carriers near counter electrode region while avoids zeroing the electric field near collection electrode region. Taking advantage of these, PCE enhancements are obtained from 15.4% to 16.2% and from 16.9% to 18.0%, respectively, via cathode p‐doping and anode n‐doping. Extending its application from opaque to semitransparent devices, the PCE of dilute cell rises from 10.5% to 12.1%, with a high light utilization efficiency (LUE) of 3.5%. The findings provide practical solutions to the core device physical problem in OSCs.

## Graphical abstract

Counter‐electrode doping strengthens the low mobility carrier collection at the far end by reshaping the internal electric potential distribution within the active layer. Applying this strategy to semitransparent devices, a good balance between PCE and AVT is achieved to produce a higher LUE.

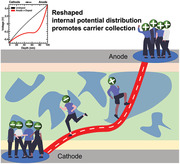

## 1 Introduction

The rapid developments on material design of OSCs have led to renewal PCE records approaching 20% in recent years.^[^ ^1^, ^2^ ^]^ However, the material‐driven PCE growth has encountered a bottleneck since it has reached the status with a negligible donor/acceptor energy offset at heterojunction^[^ ^3^, ^4^ ^]^ and an optical bandgap being close to the optimal Shockley–Queisser bandgap in state‐of‐the‐art material combinations.^[^ ^5^, ^6^ ^]^ In spite of the excellent optical properties of wide absorption range and high extinction coefficient, the poor electric properties of OSC materials, in particular, the low and imbalanced carrier mobility is considered as the main reason for the lagged PCE value compared to the inorganic and hybrid perovskite solar cells.^[^ ^7^, ^8^ ^]^ The strong electron‐phonon coupling together with the energetic and structure disorder determines the hopping mode polaron transport in OSC materials, which restricts the electron and hole mobility on the order of 10^−5^–10^−4^ cm^2^ Vs^−1^ in most donor/acceptor bulk heterojunction (BHJ) systems.^[^ ^7^, ^9^, ^10^ ^]^ Moreover, the free holes and electrons transport occurs separately through the donor and acceptor pathways, where the two materials with various contents and morphologies inevitably display different carrier mobilities. Usually, one is several times larger than the other. The imbalanced carrier mobility, especially the lower mobility, hinders efficient charge collection far from the corresponding electrode at low internal electric field. Therefore, the BHJ film thickness and the donor/acceptor ratio are restricted to be ≈100 nm with 1:1 ratio, which are however unsuitable for commercial utilization such as printable colorful and semitransparent OSCs (ST‐OSCs).

Although the unipolar charge mobility can be enhanced to 10^−2^ cm^2^ Vs^−1^ by designing rigid conjugated backbone,^[^ ^11^ ^]^ the requirement on forming optimal BHJ morphology makes it impossible to simultaneously enhance the bipolar charge transporting ability. Hence, merely enhancing the mobility of one particular carrier aggravates the imbalanced charge transport situation, and may lead to even poorer OSC performance. Electrical doping can increase the charge mobility through providing more hopping sites and passivating traps in model cases.^[^ ^12^, ^13^, ^14^ ^]^ However, in some cases, BHJ doping results in compromised device performance, especially short‐circuit current (*J*_SC_) and fill factor (FF).^[^ ^15^, ^16^ ^]^ Adding certain types of additives has been proven to improve the charge mobilities, yet this is materials sensitive.^[^ ^2^, ^6^, ^17^, ^18^ ^]^ Alternative to forming a uniform BHJ morphology, sequentially depositing the donor and acceptor layers forms the vertical component distribution.^[^ ^6^, ^19^ ^]^ It is believed that more donors and acceptors close to the anode and cathode respectively, would benefit for charge collection, because of the reduced charge transporting distance to electrode. However, the pseudo bilayer structure compromises between the exciton splitting area and charge transporting distance. Therefore, it usually leads to comparable PCE value to that of the BHJ device in most high‐efficiency systems.

Given the fact that the charge mobility is not uniform, an effective means to “push” the charge transport faster is by increasing the electric field. To enhance the internal electric field, an intuitive idea would be to increase the device built‐in potential. Unfortunately, it is very difficult to improve the built‐in potential due to the Fermi‐level pinning effect. For organic semiconductors, although applying contacts with extreme work function (far beyond Fermi‐level pinning) can increase the built‐in potential, the increment is however small due to its intrinsic disordering‐induced soft Fermi‐level pinning. For example, in the poly(benzo[1,2‐b:4,5‐b′]dithiophene–thieno[3,4‐c]pyrrole‐4,6‐dione) (PBDTTPD):[6,6]‐phenyl C61‐butyric acid methyl ester (PC_61_BM) system, even when contact work function is 1 eV beyond the Fermi‐level, the built‐in potential increment is only 0.05 eV, leading to merely ≈5% internal electric field increment.^[^ ^20^ ^]^ Under thermal equilibrium condition, the internal electric field is uniformly distributed. The slight built‐in potential increment does not strongly alter the electric field distribution, thus this only provides a very limited effect on the low mobility carriers transport at the far end. At present, developing a strategy to address the imbalanced mobility‐induced charge extraction remains a key challenge in OSCs.

To address this core challenge, we propose a counter‐electrode doping strategy to enhance the low mobility carrier extraction via reshaping the internal electric field. We first conducted classical drift‐diffusion‐generation (DDG) simulation by anode *p‐*doping and cathode *p‐*doping, respectively, with systematically increased doping depth. Interestingly, we observed that the cathode *p‐*doping at a certain depth improved the FF by strengthening the electric field near cathode. Setting a series of hole mobility (μ_h_)/ electron mobility (μ_e_) ratios, we found that the counter‐electrode doping strategy, with a partially doped active layer, was more effective for unbalanced mobilities. Our device results of cathode *p*‐doping and anode *n*‐doping well supported the simulated phenomena. We further explored this strategy to ST‐OSCs, where the dilute‐component active layer was believed to suffer from severe imbalanced mobility‐led charge recombination. The LUE increased from 3.1% to 3.5% due to PCE growth. The exciting performance enhancements in opaque and semitransparent devices demonstrated our counter‐electrode doping strategy as a powerful tool to overcome the imbalanced carrier mobility limitations in future OSC developments.

## 2 Results and Discussion

### 2.1 Device Physics Simulation

**Figure 1:**
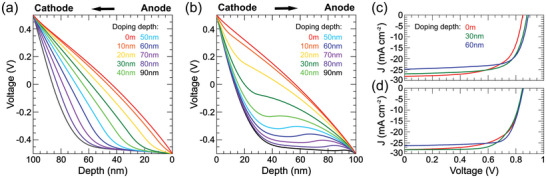
Simulated electric potential profile at J_SC_ condition: a) *p*‐doping at anode and b) *p*‐doping at cathode. The hole mobility is *μ*_h_ = 1 × 10^−4^ cm^2^ Vs^−1^, electron mobility is *μ*_e_ = 1 × 10^−3^ cm^2^ Vs^−1^. Simulated *J*–*V* characteristics of c) *p*‐doping at anode and d) *p*‐doping at cathode. The hole mobility is *μ*_h_ = 1 × 10^−4^ cm^2^ Vs^−1^, electron mobility is *μ*_e_ = 1 × 10^−3^ cm^2^ Vs^−1^ with different doping depth. Doping density is 2  × 10^17^ cm^−3^. The doping depth increases at a step of 10 nm from red line to black line. Other DDG parameters: built‐in potential: 1.0 V; geminate recombination rate: 10^4^ s^−1^; contact carrier density: 2 × 10^17^ cm^−3^.

From basic physics, we know that the electric doping and dopant distribution affect the internal potential and corresponding electric field within a device. Previous studies have demonstrated that electric doping induced large internal electric field bending.^[^ ^21^, ^22^ ^]^ Although the large internal electric field bending improves charge transport within this region, it severely hinders charge transport at the low electric field region. Consequently, it worsens charge recombination and lowers *J*_SC_ and FF.^[^ ^23^, ^24^ ^]^ Taking a common BHJ blend with unbalanced charge carrier mobility as an example, i.e., μ_h_ is lower.^[^ ^25^ ^]^ In this case, the optimal internal electric field condition would be to improve the hole transport at “long transport distance”, that is, the electric field should be larger near the cathode region. Even though the electric field near the anode region will be consequently smaller, this only has a minor effect on the hole transport. Traditionally, the BHJ doping refers to uniform doping along the whole active layer; or even at some cases the film is partially doped, the doping strategy is *p‐*doping at anode and *n‐*doping at cathode. These strategies lead to severely more carriers at the corresponding contact, and thus “flattened” electric potential, as indicated in **Figure** **1**a. On the questions of how *p‐*dope at cathode and/or *n‐*dope at anode would affect the internal electric field and consequent device performance have yet been tested.

To systematically investigate the effect of anode *p‐*doping and cathode *p‐*doping on solar cell performance, we first simulated the electric potential profile at short‐circuit condition (details in Supporting Information). We adopt the classic drift‐diffusion‐generation (DDG) model with doping being considered, as proposed by Koster et al. and Stelzl et al.^[^ ^26^ ^]^ As shown in Figure S1 (Supporting Information), we obtained similar results using similar device parameters, which confirmed the validity of our model. The calculated electric potential profile at a *p*‐doping level of 2 × 10^17^ cm^−3^ with different doping depths from anode and cathode are shown in Figure 1a,b, respectively. Without doping, the internal potential is practically uniform, which becomes non‐uniform after *p*‐dopant addition. For anode *p‐*doping with low doping depth (<30 nm), the electric field at doped region is approaching to zero as doping depth increases, while which at un‐doped region increases accordingly by ≈20% (Figure 1a). Although further increasing the doping depth to whole layer strengthens the electric field by ≈100% near cathode region, this results in near zero electric field at 50 nm from the anode. Such a large zero electric field region will reversely affect the electron and hole transport. Therefore, the traditional anode *p‐*doping strategy can hardly improve the device performance. Interestingly, for cathode *p‐*doping, an S‐shaped electric potential appears as doping depth increases. A larger electric field immediately achieves near the cathode while maintaining a relatively considerable electric field near the anode region (Figure 1b). Despite the slightly decreased electric field in the middle region, the S‐shaped electric potential profile guarantees sufficient electric field at both of anode and cathode. Apparently, this electric field distribution is more favorable for carrier extraction as compared to the anode *p‐*doping strategy.

The simulated *J*‐*V* characteristics of the anode and cathode *p‐*doping devices with different doping depths (0, 30, and 60 nm) are shown in Figure 1c,d, respectively. The doping level is 2 × 10^17^ cm^−3^, and other DDG simulation parameters are listed in the caption of Figure 1 and Table S1 (Supporting Information). Without doping, the *V*_OC_ is 0.850 V, *J*_SC_ is 28.1 mA cm^−2^, FF is 64.8%, and PCE is 15.5%. For anode *p‐*doping with a doping depth of 30 and 60 nm, the *V*_OC_ increases by 0.03 V and 0.04 V, *J*_SC_ continuously reduces to 27.0 and 24.9 mA cm^−2^, and FF increases to 69.7% and 72.0%, correspondingly. Hence, the PCE first increases to 16.5% and then decreases to 15.9% (Figure 1c). On the other hand, for cathode *p‐*doping with doping depth of 30 nm and 60 nm, the *V*_OC_ only slightly increases by <0.005 V. The *J*_SC_ first keeps practically unchanged and then slightly decreases to 26.4 mA cm^−2^. FF significantly increases to 69.2% and 74.7%, correspondingly. The FF increment dominates PCE continuously increasing to 17.1% and 16.8%, respectively (Figure 1d). Note that in our model, the charge‐carrier mobility is set to be independent of the doping concentration in order to determine the effect of each parameter independently. In fact, considering a hole‐density‐dependent‐mobility does not change the results (Figure S2, Supporting Information).

**Figure 2:**
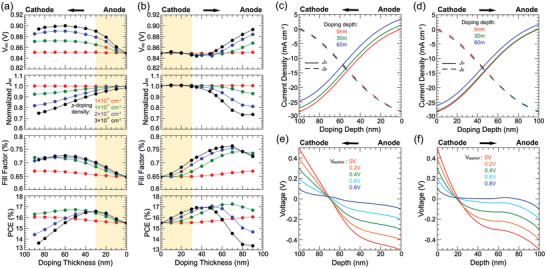
Simulated device performance of *p*‐doping at a) anode and b) cathode with different doping depths and doping densities. Simulated electron current and hole current profiles of *p*‐doping at c) anode and d) cathode with different doping depths. Simulated electric potential profiles of *p*‐doping at e) anode and f) cathode with different applied voltages, doping density is 10^17^ cm^−3^, doping depth is 60 nm. *μ*_e_ = 1 × 10^−3^ cm^2^ Vs^−1^, *μ*_h_ = 1 × 10^−4^ cm^2^ Vs^−1^. *V*_bi_ = 0.9 V, *k*_f_ = 10^3^ s^−1^.

To further illustrate the doping depth dependent device performance, we conducted systematic DDG simulations with doping levels of 1 × 10^16^, 1 × 10^17^, 2 × 10^17^, and 3 × 10^17^ cm^−3^, and doping depth within the range of 0 to 90 nm. The simulation results are summarized in **Figure** **2**a,b. At doping depth <40 nm, the *V*_OC_ of cathode *p‐*doped devices remains unchanged, while the anode *p‐*doped devices show clearly improved *V*_OC_. This is because cathode *p‐*doping does not induce significant band‐bending at neither anode nor cathode as indicated in Figure 2c,d. Thereby it has limited effects on reducing the injection current, which improves *V*_OC_. These phenomena are consistent with the literature reports where *p‐*doping in the whole film could improve *V*_OC_.^[^ ^26^, ^27^, ^28^ ^]^ Interestingly, the *J*_SC_ shows different trends. For cathode *p‐*doping with shallow doping depth, the *J*_SC_ does not suffer from doping. However, for the case of anode *p‐*doping, *J*_SC_ readily starts to reduce at shallow doping depth. For instance, at 40 nm doping depth with *N*_p_ = 2 × 10^17^ cm^−3^, the *J*_sc_ of anode *p‐*doped device reduces by 6.6%, while the cathode *p‐*doped device only suffers from *J*_SC_ reduction of <1%. Further increasing the doping level or doping depth will cause more losses in *J*_SC_ for both doping cases, eventually to over 20% at 90 nm doping depth. Therefore, the doping depth should be limited nearby the cathode to avoid significant *J*_SC_ loss. These results show that the loss in *J*_SC_ is mainly due to the “leak” of electron crrent (*J*_n_) at anode, as indicated in Figure 2c,d. The hole current (*J*_p_) is almost unaffected by doping despite the redistribution of electric field, while *J*_n_ is sensitive to the doping depth. As doping depth or doping level increases, the *J*_n_ at anode becomes positive. Since doping “flattens” the electric field at anode, the electron tends to flow out from anode driven by concentration gradients. The leak current leads to the positive *J*_n_ at anode and thus decreases photocurrent (and *J*_SC_). Applying selective contact where *J*_n_ at anode is enforced to zero will effectively address the electron leak current issue, as shown in Figure S3 (Supporting Information).

In addition to *V*_OC_ and *J*_SC_ improvements, we also noticed that cathode *p‐*doping is more favorable for carrier extraction. The FF improves more for cathode *p*‐doped devices than for anode *p‐*doped devices with the same doping level and depth. This could be understood through the electric potential profile at different bias voltages, as presented in Figure 2e,f. As bias increases from 0 to 0.8 V, the electric potential of cathode *p*‐doped device is more balanced compared to the anode *p*‐doped device. Therefore, the cathode *p‐*doping supports more efficient charge carrier transport even at low internal electric field. The *V*_OC_ of the anode‐doped device gradually reaches saturation as the doping depth increases, while the *V*_OC_ of the cathode *p‐*doped device begins to increase after the dopant enters the anode side, and eventually, the *V*_OC_ of the two devices is equal. Since the electric potential of cathode *p‐*doped device is now becoming alike to the anode *p‐*doped device, its *J*_SC_ also starts to decrease rapidly. Simultaneously, the FF also decreases at deep doping depth (roughly >60 nm), primarily due to the low electric field induced large recombination nearby anode region (50–100 nm). In spite of the opposite behaviors on *J*_SC_ and FF as compared to the un‐doped device, we still obtain the highest cathode *p‐*doping PCE of 17.1% and anode *p‐*doping PCE of 16.5% at a doping level of *N*_p_ = 2 × 10^17^ cm^−3^ with 30 nm doping depth (Table S1, Supporting Information). We also simulated the doping effect of device with different carrier mobility sets, as shown in the Figures S4–S8 (Supporting Information). For cathode *p‐*doping with high *μ*_h_, a smaller improvement in FF and a similar PCE can be obtained for cathode *p‐*doped devices compared to low *μ*_h_ devices as *μ*_h_ keeps increasing closer to *μ*_e_ (Table S2, Supporting Information). This is because the *p‐*doping strategy results larger electric field far from the anode, thus it is more effective for hole extraction in the low *μ*_h_ devices. Therefore, for photoactive materials with relatively low *μ*_h_, the cathode *p‐*doping strategy provides a powerful method to improve the device performance.

### 2.2 Cathode p‐Doped OSCs

**Figure 3:**
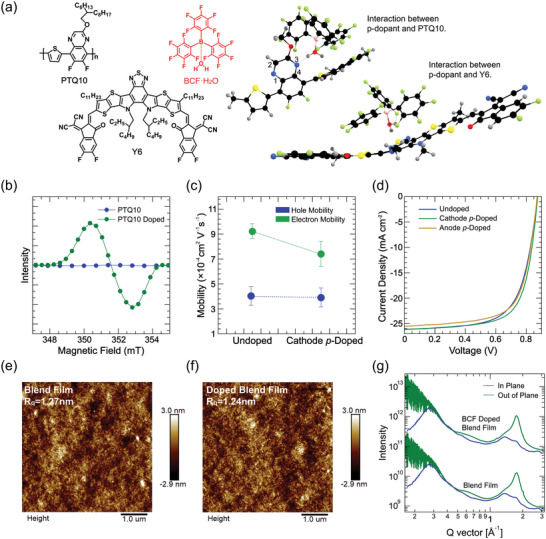
a) The chemical structures and estimated interactions calculated by DFT calculation of PTQ10, Y6 and BCF·H_2_O. b) ESR spectra of PTQ10. c) Hole and electron mobilities before and after cathode *p‐*doping, the average parameters are calculated from ten devices. d) *J*–*V* curves of the undoped and BCF (0.01 mg mL^−1^) doped devices based on PTQ10:Y6 (1:1.2) with a device area of 4 mm^2^. (e) and (f) AFM height images of undoped and BCF doped samples based on PTQ10 and PTQ10:Y6 (1:1.2) blended films. g) The in‐plane (IP) and out‐of‐plane (OOP) line cut profiles data based on BCF doped and undoped PTQ10:Y6 blended films.

To validate the counter electrode doping strategy, we fabricated the doped OSC devices under the guidance of above simulations. We used the conventional device structure of ITO/PEDOT:PSS/Active layer/PDINO/Ag. Hydrated tris(pentafluorophenyl) borane (BCF·H_2_O, **Figure** **3**a) was chosen as bulky *p‐*dopant.^[^ ^29^, ^30^ ^]^ A polymer donor poly((6,7‐difluoro((2‐hexyldecyl)oxy)−5,8‐quinoxalinediyl)−2,5‐thiophenediyl) (PTQ10) together with 2,2′‐((2Z,2′Z)‐((12,13‐bis(2‐ethylhexyl)−3,9‐diundecyl‐12,13‐dihydro‐[1,2,5]thiadiazolo[3,4‐e]thieno[2″,3″: 4′,5′]thieno[2′,3′:4,5]pyrrolo[3,2‐g]thieno[2′,3′:4,5]thieno [3,2‐b]indole‐2,10‐diyl)bis(methaneylylidene))bis(5,6‐difluoro‐3‐oxo‐2,3‐dihydro‐1H‐indene‐2,1diylidene))dimalononitrile (Y6) was adopted as the active layer materials (Figure 3a).^[^ ^31^, ^32^ ^]^ As an important assumption, the cathode *p‐*doping performs better in material combinations with lower hole mobility. In accordance with it, we examined the carrier mobility in PTQ10:Y6 BHJ film at optimum donor:acceptor (D:A) weight ratio of 1:1.2. In Figure 3c and Figure S9 (Supporting Information), we observed lower μ_h_ of (4.52 ± 0.10) × 10^−4^ cm^2^ Vs^−1^ than *μ*_e_ of (9.24 ± 0.12) × 10^−4^ cm^2^ Vs^−1^. In an ideal case, it is also expected that the BCF·H_2_O should selectively dope PTQ10 rather than Y6. Density functional theory (DFT) simulations and electron‐spin resonance (ESR) measurements examined the doping selectivity. The former provides information on the H^+^ transfer from dopant to PTQ10, and the latter provides information on forming the radical product.^[^ ^30^, ^33^, ^34^ ^]^ The O atom of the water molecule binding to the B atom of the *p‐*dopant BCF causes the bond lengths of the H‐O of the water to increase to 0.9728 Å at point 1 and 0.9735 Å at point 2 (Figure S10, Supporting Information). This result favors the H^+^ transfer to PTQ10 via breaking of the H‐O bond of the water molecule. Using a dimer to represent for PTQ10, the length of the isolated polymer bonds at points 1, 2, 3 and 4 are: 1.350 Å at point 1, 1.306 Å at point 2, 1.304 Å at point 3 and 1.350 Å at point 4 (Figure S11, Supporting Information). When the ring of the dimer containing N atom comes into contact with the BCF and with the water, coordination takes place between the H atoms of the water, the F atom of the BCF and the N atom of the polymer. The coordination causes an increase in bond length at point 3 to 1.310 Å and at point 4 to 1.360 Å (Table S3, Supporting Information). The bond lengths of O─H in BCF·H_2_O contained water also increase from 0.9735 to 1.006 Å in contact with the dimer (Figure 3a). When we put the Y6 in contact with the BCF and with the water, we notice a twist in the chemical structure of Y6; however, there is no interaction between the H atom in the water and the N atom in Y6. The length of the bonds between H and atoms in the water molecule does not change (Figure S12, Supporting Information). The ESR measurements also support the selective doping effect of BCF·H_2_O on PTQ10. We observe the polaron signals in *p‐*doped PTQ10 film, whereas it is absent in Y6 film (Figure 3b; Figure S13, Supporting Information).

Having chosen the paradigm materials, we then examined the cathode *p‐*doping strategy in real devices. The doping depth in the active layer depends on the material‐solvent miscibility, which we evaluated by the Flory‐Huggins interaction parameters (Table S4, Supporting Information).^[^ ^35^ ^]^ We determined the doping depth by varying the ratio of acetonitrile to ethanol in the solvent mixture. The dopant is dissolved in a mixture of acetonitrile at 100%, 50%, and 0%, respectively, and Time of Flight Secondary Ion Mass Spectrometry (TOF‐SIMS) tests is used to reveal the actual doping depths in the three conditions. In depth profiling test, cyano is used as a characteristic fragment to trace Y6 and Boron is used as a characteristic element to trace the BCF·H_2_O distribution in the vertical direction of active layer. The depth profile obtained is shown in Figure S14 (Supporting Information). The dashed lines represent the distribution of Y6, and the active layer thickness can be judged to be ≈100 nm. The solid lines show the distribution of BCF·H_2_O. The maximum doping depth is ≈45 nm when using pure acetonitrile as the solvent, which is about half of the active layer thickness, as shown by the red solid line in Figure S14 (Supporting Information). This is very close to the optimal doping depth obtained from our simulations. The maximum doping depth decreases with increasing ethanol content. Under the condition of pure ethanol dopant solution, the dopant basically distribute only in the surface layer of the active layer, as shown by the blue solid line in Figure S14 (Supporting Information). Therefore, we finally choose pure acetonitrile as the dopant solvent. Acetonitrile also has a high permittivity of 36.7, which facilitates the doping reaction by reducing the Coulombic attraction between the transferred electron and its counter ion.^[^ ^36^ ^]^ Details of the device fabrication and photovoltaic performance tests are given in the Supporting Information. The *J*–*V* photovoltaic performances of the devices with a certain depth of cathode *p‐*doping are shown in Figure 3d, **Table** **1**, and Table S5 (Supporting Information). The undoped device has a maximum PCE of 15.4% with FF of 67.5%, *J*_SC_ of 26.3 mA cm^−2^, and *V*_OC_ of 0.878 V. After spin‐coating 0.01 mg mL^−1^ BCF·H_2_O solution on top of the photoactive layer, the PCE increases to 16.2%, in which the FF increment of 70.9% dominates the *p‐*doping contribution. Though the charge mobility changes a little bit after cathode *p‐*doping, the average *μ*_h_ of (4.57  ± 0.08) × 10^−4^ cm^2^ Vs^−1^ is still a bit lower than the average *μ*_e_ of (7.46 ± 0.20) × 10^−4^ cm^2^ Vs^−1^ (Figure S9, Supporting Information). As analyzed in our theoretical descriptions, the enhanced mobility is not the reason for better OSC performance, which contradicts the common sense of doping effect. Furthermore, we fabricated devices with an inverted structure of ITO/ZnO/Active Layer/MoO_3_/Ag to examine the anode *p‐*doping influences on photovoltaic performance. As predicted, the anode *p‐*doping increases the FF to 69.4% and *V*_OC_ to 0.880 V. However, the *J*_SC_ drops to 25.7 mA cm^−2^, which has been explained by the *J*_n_ enhancement due to leak current (Figure 2). The variation of current density is further confirmed by the external quantum efficiency (EQE) measurements, which give the integrated *J*_SC_ of 25.9, 26.0, and 25.4 mA cm^−2^ in the undoped, cathode *p‐*doped, and cathode *n‐*doped devices, respectively (Table 1; Figure S15, Supporting Information). The PCE of 15.8% by anode *p*‐doping is inferior to that of cathode *p*‐doping. To exclude the possibility of total built‐in electric field change for the better device performance, we measured the built‐in potential (*V*_bi_) by analyzing the photocurrent (*J*_L_) and dark current (*J*_D_) curves under various applied voltages (*V*_a_). The voltage value (*V*_bi_) at which *J*_L_‐*J*_D_ = 0 was determined. Before and after doping, *V*_bi_ was calculated to be 0.91 V for the PTQ10:Y6 (Figure S16, Supporting Information).**^[^** ^37^ ^]^ In addition, the cathode p‐doped devices facilitate better stability in comparison to those undoped devices. The dark aging condition stabilities of the undoped and doped OSCs are taken by tracking the unencapsulated device parameters for over 1000 h in N_2_ glovebox. The cathode *p*‐doped devices exhibit a slight PCE decease and retain ≈90% of its original PCEs after 1000 h storage at room temperature (RT). Furthermore, the undoped devices suffer from ≈20% degradation of its initial PCEs during the same intervals (Figure S17, Supporting Information). In addition, we also evaluated the operational stability of the devices by tracking the maximum power point (MPP) at 100 mW cm^−2^ light.^[^ ^38^, ^39^ ^]^ As shown in Figure S18a (Supporting Information), both undoped and cathode p‐doped PTQ10:Y6 devices maintained about 74% of the initial PCE after aging for 48 h at 1 sunlight.

**Table 1:** Photovoltaic parameters of undoped, cathode *p*‐doped, and anode *p*‐doped devices based on PTQ10:Y6 (1:1.2) with a device area of 4 mm^2^.^a)^

Condition	*V*_OC_ [V]	*J*_SC_ [mA cm^−2^]	FF [%]	PCE [%]	EQE [mA cm^−2^]
Control	0.878 (0.877 ± 0.001)	26.3 (26.0 ± 0.1)	67.5 (67.2 ± 0.3)	15.4 (15.3 ± 0.1)	25.9
Cathode *p*‐doped	0.878 (0.876 ± 0.002)	26.3 (26.1 ± 0.1)	70.9 (70.3 ± 0.2)	16.2 (16.1 ± 0.1)	26.0
Anode *p*‐doped	0.880 (0.879 ± 0.001)	25.7 (25.4 ± 0.2)	69.4 (69.7 ± 0.5)	15.8 (15.5 ± 0.1)	25.4

^a)^ The average PCEs are obtained from ten devices.

To rule out the morphology variations in photoactive layer upon doping that lead to the device performance improvement, we evaluated the influences of cathode *p‐*doping on blend film morphology by tapping‐mode atomic force microscopy (TM‐AFM) and grazing incident wide angle X‐ray scattering (GIWAXS) measurements. The AFM images show none of geometric morphology change on the surface of PTQ10:Y6 blend film after sequential BCF·H_2_O coating due to the slight surface roughness change from 1.27 to 1.24 nm (Figure 3e,f). Structure parameters from the GIWAXS two‐dimensional (2D) diffraction patterns (Figure S19, Supporting Information) and one‐dimensional (1D) line cuts (Figure 3g) are summarized in Table S6, where the subtle variations in (100), (111), and (010) d‐spacing distances and coherence lengths (CLs) cannot explain the device improvement. These results can rule out the morphology artefacts and point out that the electronic doping can independently optimize OSCs through electric mechanism.

### 2.3 Double‐BHJ‐Layer Model OSCs

**Figure 4:**
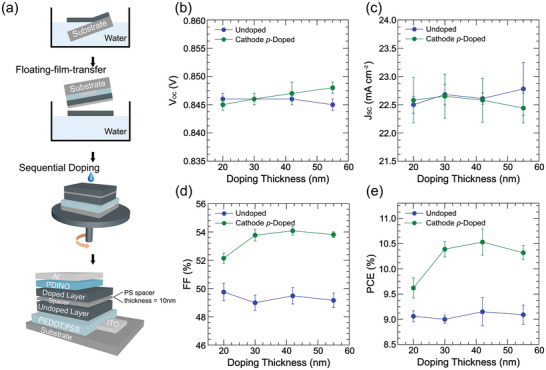
a) Schematic showing the process of fabricating a double‐BHJ‐layer (PTQ10:Y6 = 1:1.2) device. (b) *V*_OC_, c) *J*_SC_, d) FF, and e) PCE plots of double‐BHJ‐layer devices at undoped and cathode p‐doped conditions as a function of doping depth. The parameters are obtained from >10 independent devices.

The fabrication of single‐BHJ‐layer OSCs successfully proves the predicted benefits of cathode *p‐*doping in real devices, however, the *p‐*dopant diffusion depth is unknown. To provide a complete evidence chain of its influences on photovoltaic performance, herein, we prepared double‐BHJ‐layer (PTQ10:Y6 = 1:1.2) devices with confined *p‐*doping depths to demonstrate the depth‐dependent device properties. The confined doping depth is achieved by inserting a 10 nm dopant blocking layer of high molecular weight (198 kDa) polystyrene (PS) between two thickness‐variable BHJ films (Figure S20, Supporting Information).^[^ ^40^ ^]^ The device fabrication process is displayed in **Figure** **4**a, and the experimental details are described in Supporting Information.^[^ ^16^, ^41^ ^]^ By tuning the film thicknesses of top and bottom BHJ layers, we obtain four doping depths of ≈20, ≈30, ≈42, and ≈55 nm within a total active layer thickness of ≈85 nm (excluding the PS interlayer). The device performance with depth dependent photovoltaic parameters is summarized in Figure 4b–e and Table S7 (Supporting Information). Since the device parameters fluctuate in various combinations of top and bottom BHJ layer thickness, we evaluate the doping influence on each photovoltaic parameter by using the offsets in corresponding values between the cathode *p*‐doped and undoped devices. The small *V*_OC_ increment of <0.005 V is observed when the doping depth is over half of the active layer thickness (Figure 4b), which is consistent with the device simulations. The *J*_SC_ offset is insensitive at shallow doping depth below ≈42 nm, and starts to decrease at ≈55 nm doping depth (Figure 4c). As discussed in the simulation section, the *J*_SC_ decreases when an undesirable dopant distribution in the anode side disrupts the balanced potential within the device. Though the FF values significantly reduce by inclusion of the PS blocking layer from over 67% in single‐layer devices to <50% in double‐BHJ‐layer devices, the FF enhancement trend is still observed in all doped devices (Figure 4d). Looking into the overall influences of cathode *p‐*doping on PCE values, its increment initially enlarges and then drops at doping depth of 55 nm due to the reduced *J*_SC_ (Figure 4e). Compared to the undoped device (*V*_OC_ = 0.840 V, *J*_SC_ = 22.6 mA cm^−2^, FF = 49.5%, and PCE = 9.2%), the highest PCE of 10.5% is obtained at a doping depth of ≈42 nm (*V*_OC_ = 0.840 V, *J*_SC_ = 22.6 mA cm^−2^, FF = 54.1%). The double‐BHJ‐layer model devices support the simulation results very well, and validate the effectiveness of the counter electrode doping strategy.

### 2.4 Anode n‐Doped OSCs

**Figure 5:**
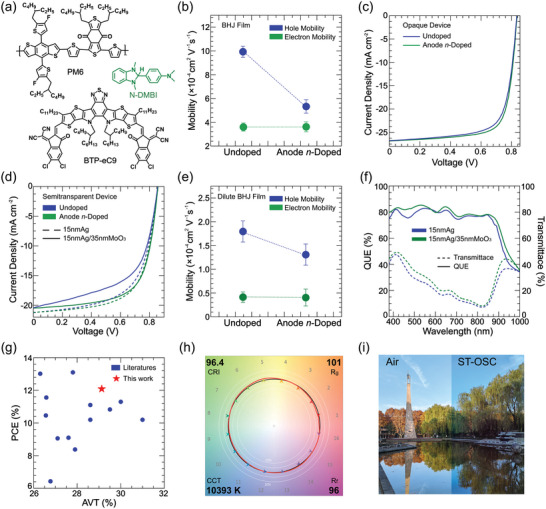
a) The chemical structures of PM6, BTP‐eC9 and N‐DMBI. b) Hole and electron mobilities of PM6:BTP‐eC9 BHJ devices before and after anode n‐doping. c) *J*–*V* curves of the pristine and N‐DMBI (0.01 mg mL^−1^) anode n‐doped BHJ devices based on PM6:BTP‐eC9 (1:1.2). d) *J*–*V* curves of the pristine and N‐DMBI (0.01 mg mL^−1^) anode n‐doped semitransparent devices based on PM6:BTP‐eC9 (1:3) with and without ARC layer. e) Hole and electron mobilities of PM6:BTP‐eC9 dilute devices before and after anode n‐doping. The parameters are obtained from ten independent devices. f) The quantum utilization efficiency (QUE) and transmittance curves of ST‐OSC with and without ARC layer. g) Comparison of our result with the AVT reported in the literatures without complex optical engineering. h) The Color Vector Graphic (CVG) of the optimal device shows the average change in a',b') coordinates of CAM02‐UCS for the CES within each hue‐angle bin. (Background is for visual orientation only) i) Photographs of landscape viewed through air and ST‐OSC device.

In parallel to cathode *p‐*doping, we further extended our strategy to the anode *n‐*doping case. We chose poly[(2,6‐(4,8‐bis(5‐(2‐ethylhexyl‐3‐fluoro)thiophen‐2‐yl)‐benzo[1,2‐b:4,5‐b′]dithiophene))‐alt‐(5,5‐(1′,3′‐di‐2‐thienyl‐5′,7′‐bis(2‐ethylhexyl)benzo[1′,2′‐c:4′,5′‐c′]dithiophene‐4,8‐dione)] (PM6):2,2′‐((2Z,2′Z)‐((12,13‐bis(2‐butyloctyl)−3,9‐dinonyl‐ 12,13‐dihydro‐[1,2,5]thiadiazolo[3,4‐e]thieno[2″,3″:4′,5′]thieno[2′,3′:4,5]pyrrolo[3,2‐g]thieno[2′,3′:4,5]thieno[3,2‐b]indole‐2,10‐diyl)bis (methaneylylidene))bis(5,6‐dichloro‐3‐oxo‐2,3‐dihydro‐1H‐indene‐2,1‐diylidene))dimalononitrile (BTP‐eC9) as the testbed (**Figure** **5**a), because the average μ_e_ ((3.62 ± 0.05) × 10^−4^ cm^2^ Vs^−1^) is lower than μ_h_ ((9.96 ± 0.25) × 10^−4^ cm^2^ Vs^−1^) in optimal BHJ blend film (Figure 5b; Figures S21 and S25, Supporting Information).^[^ ^17^, ^42^ ^]^ (4‐(1,3‐dimethyl‐2,3‐di‐hydro‐1H‐benzoimidazol‐2‐yl)phenyl)dimethylamine (N‐DMBI) is taken as the n‐dopant, and spin‐coated on top of an inverted device (ITO/ZnO/Active layer/MoO_3_/Ag).^[^ ^43^ ^]^ To provide evidence of N‐DMBI doping in BTP‐eC9, we conducted DFT simulations and ESR spectroscopy measurement. DFT simulations reveal information on the charge transfer between the n‐dopant N‐DMBI and the acceptor BTP‐eC9. The simulations show that the removal of H atom from N‐DMBI is the first step in the doping process, while free protons are free to interact with the n‐dopant ion or the acceptor. In order to find the most likely doping mechanism, we calculate the amount of charge transfer of the two interactions. A small amount of charge transfer occurs when the free proton is coordinated to the sulfur atom in the acceptor thiophene ring, which is only 0.458 *e*. However, when the free protons coordinate with the carbon atoms of the dopant, the bond lengths at points 6 and 7 of N‐DMBI increase from 1.38 and 1.39 Å to 1.50 and 1.52 Å, respectively. Then the doped system interacts with the acceptor, which shows an almost integer electron transfer of 0.940 *e* (Figure S22 and Tables S8 and S9, Supporting Information). By ESR testing, we observed polarization signals in n‐doped BTP‐eC9 films, which provide evidence for n‐doping of the acceptor by N‐DMBI (Figure S23, Supporting Information). The anode *n‐*doping produces an impressive PCE improvement from 16.9% to 18.0%, which mainly results from an FF enhancement from 72.7% to 76.7% (Figure 5c and **Table** **2**; Figure S24 and Table S10, Supporting Information). After anode *n‐*doping, the average μ_h_ declines to (5.02 ± 0.30) × 10^−4^ cm^2^ Vs^−1^, while the average μ_e_ retains to (3.59 ± 0.01) × 10^−4^ cm^2^ Vs^−1^ (Figure 5b). The remaining imbalanced carrier mobility emphasizes that the doping role on better FF is not changing the mobility as predicted and observed in the cathode *p‐*doping case. As the situation in cathode p‐doping, the built‐in potential change was not the reason for better device performance (Figure S16, Supporting Information). More importantly, the anode *n*‐doped devices show an amazing RT storage stability, maintaining 98% PCE of its initial PCEs after 1000 h dark aging in N_2_ glovebox. While the performance of the undoped devices drops faster under the same condition as shown in Figure S17 (Supporting Information). Although the photovoltaic devices are manufactured in a glove box, they are still not immune to the effects of water and oxygen. Benefiting from molecular doping that passivates water‐oxygen defects and hinders the diffusion of water and oxygen in the active layer, the doped devices achieve better dark stability. After soaking in one sunlight, the devices still show good stability, where 88% of the initial PCE conserved after 48 h aging (Figure S18b, Supporting Information).^[^ ^44^, ^45^, ^46^ ^]^ We extend the anode n‐doping strategy in other NFA materials, such as Y6 ((2,2′‐((2Z,2′Z)‐((12,13‐bis(2‐ethylhexyl)−3,9‐diundecyl‐12,13‐dihydro‐[1,2,5]thiadiazolo[3,4‐e]thieno[2″,3″:4′,5′]thieno[2′,3′:4,5]pyrrolo[3,2‐g]thieno[2′,3′:4,5]thieno[3,2‐b]indole‐2,10‐diyl)bis(methaneylylidene))bis(5,6‐difluoro‐3‐oxo‐2,3‐dihydro‐1H‐indene‐2,1‐diylidene))dimalononitrile)) and L8‐BO ((2,2′‐((2Z,2′Z)‐((3,9‐bis(2‐butyloctyl)−12,13‐bis(2‐ethylhexyl)−12,13‐dihydro‐[1,2,5]thiadiazolo[3,4‐e]thieno[2″,3″:4′,5′]thieno[2′,3′:4,5]pyrrolo[3,2‐g]thieno[2′,3′:4,5]thieno[3,2‐b]indole‐2,10‐diyl)bis(methaneylylidene))bis(5,6‐difluoro‐3‐oxo‐2,3‐dihydro‐1H‐indene‐2,1‐diylidene))dimalononitrile)). In Y6 and PM6 blend system, anode n‐doping can increase its FF from 69.3% to 72.7%, which in turn increases the PCE from 15.4% to 16.1% (Figure S26a and Table S11, Supporting Information). When combing the PM6 with L8‐BO, the FF increases from 73.1% to 76.9%, accompany with PCE enhancement from 16.6% to 17.5% (Figure S26b and Table S11, Supporting Information).

**Table 2:** Photovoltaic parameters of undoped, anode *n*‐doped opaque (D:A = 1:1.2) and semitransparent (D:A = 1:3) devices based on PM6:BTP‐eC9 with a device area of 4 mm^2^.^a)^

BHJ Device	*V*_OC_ [V]	*J*_SC_ [mA cm^−2^]	FF [%]	PCE [%]	EQE [mA cm^−2^]
Control	0.865 (0.862 ± 0.002)	27.0 (26.9 ± 0.1)	72.7 (71.3 ± 0.9)	16.9 (16.8 ± 0.1)	26.0
Anode *n*‐doped	0.866 (0.865 ± 0.001)	27.0 (26.7 ± 0.2)	76.7 (76.4 ± 0.3)	18.0 (17.9 ± 0.1)	26.0

Semitransparent Device		*V*_OC_ [V]	*J*_SC_ [mA cm^−2^]	FF [%]	PCE [%]	AVT [%]	LUE [%]
15 nm Ag/ 35 nm MoO_3_	Undoped	0.860 (0.859 ± 0.001)	20.7 (20.2 ± 0.1)	61.0 (60.0 ± 1.0)	10.5 (10.3 ± 0.2)	29.1	3.1
	Anode *n*‐doped	0.859 (0.858 ± 0.001)	20.7 (20.5 ± 0.1)	68.2 (67.7 ± 0.4)	12.1 (12.0 ± 0.1)		3.5

^a)^ The average PCEs are obtained from ten devices.

### 2.5 Application in ST‐OSCs

The complementary absorption range between PM6 and BTP*‐*eC9 enables them suitable for fabricating the ST‐OSC devices by diluting the PM6 content to enhance the visible light transmittance. We selected four PM6:BTP*‐*eC9 ratios (D:A = 1:2, 1:3, 1:5, 1:8) to fabricate the ST‐OSCs with device structure of ITO/ZnO/PM6:BTP*‐*eC9/MoO_3_/Ag (15 nm). The PCE decreased from 14.3% to 8.1% while the average visible transmittance (AVT) increases from 16.1% to 31.3% (Figure S27 and Table S12, Supporting Information). The opposite trends of PCE and AVT produced the highest light utilization efficiency (LUE = PCE × AVT) of 2.8% at PM6:BTP*‐*eC9 ratio of 1:3. At this condition, the average *μ*_e_ ((0.45 ± 0.05) × 10^−4^ cm^2^ Vs^−1^) is still lower than the average *μ*_h_ ((1.74 ± 0.10) × 10^−4^ cm^2^ Vs^−1^) (Figure 5e; Figure S25, Supporting Information), hence the anode *n‐*doping is adopted to enhance the ST‐OSC performance. After anode *n‐*doping by N‐DMBI for the 1:3 ST‐OSC device, the PCE increases from 11.5% to 12.3% with FF increased from 63.0% to 68.2%, while *J*_SC_ and *V*_OC_ remain unchanged (Figure 5d; Figure S28, Supporting Information). The LUE therefore raises to 3.0% (Figure S29 and Table S13, Supporting Information). The anode *n‐*doping reduces the average *μ*_h_ to (1.29 ± 0.10) × 10^−4^ cm^2^ Vs^−1^, while has no obvious influences on μ_e_ (Figure 5e). Further LUE increment is achieved by depositing a selective band pass layer of MoO_3_ (35 nm) on the 15 nm‐thick Ag electrode. Obtaining a larger AVT of 29.1% and a suppressed PCE of 12.1%, the LUE further raises to 3.5% as displayed in Figure 5f, Table 2, and Figure S29 (Supporting Information). The CIE (x, y) color coordinates of the optimal device locate at (0.278, 0.288), closing to the white light point of (0.313, 0.329) (Figure S30, Supporting Information). The quantum utilization efficiency (QUE) obtained from the sum of EQE and transmittance spectra is displayed to evaluate the efficiency from generated photons to charges and photons passing through the device (Figure 5f). We can see that the maximum QUE of the device is 85.1%, confirming the reliability of these values. This LUE represents one of the peak values of ST‐OSCs with AVT from 26% to 35% (Figure 5g; Table S14, Supporting Information), which is sufficient for commercial application as photovoltaic roof. The correlated color temperature (CCT), color rendering index (CRI), color fidelity index (*R*_f_), and color saturation gamut index (*R*_g_) are calculated to evaluate the human color perception for the anode *n‐*doped ST‐OSC device (see details in the Supporting Information). As displayed in the color vector graphic (CVG) (Figure 5h), the transmitted light source (black line) of ST‐OSC device overlaps with the reference light source (red line), and the corresponding optical values are 10 393 K, 96, 96, and 101 respectively for CCT, CRI, *R*_f_, and *R*_g_ (Figure S31, Supporting Information). All of these data indicate a small color difference when seeing through the ST‐OSC devices (Figure 5i).

## 3 Conclusion

In summary, we proposed a counter‐electrode doping strategy to overcome the imbalanced mobility‐induced charge extraction loss in high‐efficiency OSCs to achieve improved FF and overall PCE value. In contrast to the tradition uniform doping strategy or anode p‐doping strategy that results in a compromised internal electric field that hinders charge transport, cathode p‐doping with certain depth can reshape the internal electric field that is more favorable for the low mobility charge transport. Based on our counter electrode doping strategy, we successfully improve the PCE of PTQ10:Y6 devices from 15.4% to 16.2% via cathode *p‐*doping, and PM6:BTP*‐*eC9 devices from 16.9% to 18.0% via anode *n‐*doping. As far as we know, through counter electrode doping, the two systems achieved the highest PCE compared to the corresponding systems without surfactant. Our strategy can be well extended from the opaque devices to the semitransparent devices that suffer from imbalanced charge mobility, where the anode *n‐*doping significantly enhances the dilute PM6:BTP*‐*eC9 device conversion efficiency from 10.5% to 12.1%, maximizing the LUE to 3.5%. In addition to the 100 nm‐thick lab devices, we expect that this strategy could be more effective in thick film devices.^[^ ^47^ ^]^ Since the counter electrode doping strategy focuses on the electric factors, it is compatible with other OSC optimizing strategies such as material synthesis and microstructure modification. We believe this will inspire further OSC performance breakthroughs as an essential tool.

## Conflict of Interest

The authors declare no conflict of interest.

## Supporting information

Supporting Information

## Data Availability

The data that support the findings of this study are available in the supplementary material of this article.

## References

[1] K. Jiang , J. Zhang , C. Zhong , F. R. Lin , F. Qi , Q. Li , Z. Peng , W. Kaminsky , S.‐H. Jang , J. Yu , X. Deng , H. Hu , D. Shen , F. Gao , H. Ade , M. Xiao , C. Zhang , A. K. Y. Jen , Nat. Energy 2022, 7, 1076.

[2] L. Zhu , M. Zhang , J. Xu , C. Li , J. Yan , G. Zhou , W. Zhong , T. Hao , J. Song , X. Xue , Z. Zhou , R. Zeng , H. Zhu , C. C. Chen , R. C. I. MacKenzie , Y. Zou , J. Nelson , Y. Zhang , Y. Sun , F. Liu , Nat. Mater. 2022, 21, 656. 35513501 10.1038/s41563-022-01244-y

[3] A. Classen , C. L. Chochos , L. Lüer , V. G. Gregoriou , J. Wortmann , A. Osvet , K. Forberich , I. McCulloch , T. Heumüller , C. J. Brabec , Nat. Energy 2020, 5, 711.

[4] D. Qian , Z. Zheng , H. Yao , W. Tress , T. R. Hopper , S. Chen , S. Li , J. Liu , S. Chen , J. Zhang , X. K. Liu , B. Gao , L. Ouyang , Y. Jin , G. Pozina , I. A. Buyanova , W. M. Chen , O. Inganas , V. Coropceanu , J. L. Bredas , H. Yan , J. Hou , F. Zhang , A. A. Bakulin , F. Gao , Nat. Mater. 2018, 17, 703. 30013057 10.1038/s41563-018-0128-z

[5] S. Rühle , Sol. Energy 2016, 130, 139.

[6] Y. Wei , Z. Chen , G. Lu , N. Yu , C. Li , J. Gao , X. Gu , X. Hao , G. Lu , Z. Tang , J. Zhang , Z. Wei , X. Zhang , H. Huang , Adv. Mater. 2022, 34, 2204718. 10.1002/adma.202204718 35747988

[7] U. Wurfel , D. Neher , A. Spies , S. Albrecht , Nat. Commun. 2015, 6, 6951. 25907581 10.1038/ncomms7951 PMC4421856

[8] M. M. Mandoc , L. J. A. Koster , P. W. M. Blom , Appl. Phys. Lett. 2007, 90, 133504.

[9] D. Bartesaghi , C. Perez Idel , J. Kniepert , S. Roland , M. Turbiez , D. Neher , L. J. Koster , Nat. Commun. 2015, 6, 7083. 25947637 10.1038/ncomms8083 PMC4432638

[10] O. Ostroverkhova , Chem. Rev. 2016, 116, 13279. 27723323 10.1021/acs.chemrev.6b00127

[11] Z. Chen , P. Cai , J. Chen , X. Liu , L. Zhang , L. Lan , J. Peng , Y. Ma , Y. Cao , Adv. Mater. 2014, 26, 2586. 24488944 10.1002/adma.201305092

[12] Y. Tang , H. Zheng , X. Zhou , Z. Tang , W. Ma , H. Yan , Small Methods 2022, 6, 2101570. 10.1002/smtd.202101570 35138038

[13] W. Xue , Y. Tang , X. Zhou , Z. Tang , H. Zhao , T. Li , L. Zhang , S. Liu , C. Zhao , W. Ma , H. Yan , Adv. Funct. Mater. 2021, 31, 2101892.

[14] H. Yan , J. G. Manion , M. Yuan , F. P. Garcia de Arquer , G. R. McKeown , S. Beaupre , M. Leclerc , E. H. Sargent , D. S. Seferos , Adv. Mater. 2016, 28, 6491. 27171655 10.1002/adma.201601553

[15] S. Li , T. Jiang , H. Zhang , Y. Li , Q. Cheng , H. Kang , Y.‐N. Jing , L. Xiao , X. Zhang , G. Lu , Y. Zhang , H. Zhou , Sol. RRL 2023, 7, 2201011.

[16] H. Yan , Y. Tang , X. Sui , Y. Liu , B. Gao , X. Liu , S. F. Liu , J. Hou , W. Ma , ACS Energy Lett. 2019, 4, 1356.

[17] L. Guo , Q. Li , J. Ren , Y. Xu , J. Zhang , K. Zhang , Y. Cai , S. Liu , F. Huang , Energy Environ. Sci. 2022, 15, 5137.

[18] C. He , Y. Pan , Y. Ouyang , Q. Shen , Y. Gao , K. Yan , J. Fang , Y. Chen , C.‐Q. Ma , J. Min , C. Zhang , L. Zuo , H. Chen , Energy Environ. Sci. 2022, 15, 2537.

[19] L. Zhan , S. Li , X. Xia , Y. Li , X. Lu , L. Zuo , M. Shi , H. Chen , Adv. Mater. 2021, 33, 2007231. 10.1002/adma.202007231 33598972

[20] C. Zhao , C. G. Tang , Z. L. Seah , Q. M. Koh , L. L. Chua , R. Q. Png , P. K. H. Ho , Nat. Commun. 2021, 12, 2250. 33854070 10.1038/s41467-021-22358-y PMC8047006

[21] F. Deledalle , T. Kirchartz , M. S. Vezie , M. Campoy‐Quiles , P. Shakya Tuladhar , J. Nelson , J. R. Durrant , Phys. Rev. X 2015, 5, 011032.

[22] C. Liu , Z. Li , Z. Zhang , X. Zhang , L. Shen , W. Guo , L. Zhang , Y. Long , S. Ruan , Phys. Chem. Chem. Phys. 2016, 19, 245. 27901151 10.1039/c6cp07344a

[23] O. J. Sandberg , S. Dahlström , M. Nyman , S. Wilken , D. Scheunemann , R. Österbacka , Phys. Rev. Appl. 2019, 12, 034008.

[24] Y. Zhao , C. Liang , M. Sun , Q. Liu , F. Zhang , D. Li , Z. He , J. Appl. Phys. 2014, 116, 154506.

[25] F. F. Stelzl , U. Würfel , Phys. Rev. B 2012, 86, 075315.

[26] Z. Shang , T. Heumueller , R. Prasanna , G. F. Burkhard , B. D. Naab , Z. Bao , M. D. McGehee , A. Salleo , Adv. Energy Mater. 2016, 6, 1601149.

[27] N. Shintaku , M. Hiramoto , S. Izawa , J. Phys. Chem. C 2018, 122, 5248. 10.1021/acs.jpclett.8b01134 29770698

[28] H. Yan , J. Chen , K. Zhou , Y. Tang , X. Meng , X. Xu , W. Ma , Adv. Energy Mater. 2018, 8, 1703672.

[29] B. Yurash , D. X. Cao , V. V. Brus , D. Leifert , M. Wang , A. Dixon , M. Seifrid , A. E. Mansour , D. Lungwitz , T. Liu , P. J. Santiago , K. R. Graham , N. Koch , G. C. Bazan , T. Q. Nguyen , Nat. Mater. 2019, 18, 1327. 31527809 10.1038/s41563-019-0479-0

[30] C. Sun , F. Pan , H. Bin , J. Zhang , L. Xue , B. Qiu , Z. Wei , Z. G. Zhang , Y. Li , Nat. Commun. 2018, 9, 743. 29467393 10.1038/s41467-018-03207-x PMC5821836

[31] J. Yuan , Y. Zhang , L. Zhou , G. Zhang , H.‐L. Yip , T.‐K. Lau , X. Lu , C. Zhu , H. Peng , P. A. Johnson , M. Leclerc , Y. Cao , J. Ulanski , Y. Li , Y. Zou , Joule 2019, 3, 1140.

[32] T. Bathe , C. D. Dong , S. Schumacher , J. Phys. Chem. A 2022, 126, 2075. 35324192 10.1021/acs.jpca.1c09179

[33] P. S. Marques , G. Londi , B. Yurash , T. Q. Nguyen , S. Barlow , S. R. Marder , D. Beljonne , Chem. Sci. 2021, 12, 7012. 34123329 10.1039/d1sc01268a PMC8153436

[34] Y. Tang , B. Lin , H. Zhao , T. Li , W. Ma , H. Yan , ACS Appl. Mater. Interfaces 2020, 12, 13021. 32081012 10.1021/acsami.9b21252

[35] I. E. Jacobs , Y. Lin , Y. Huang , X. Ren , D. Simatos , C. Chen , D. Tjhe , M. Statz , L. Lai , P. A. Finn , W. G. Neal , G. D'Avino , V. Lemaur , S. Fratini , D. Beljonne , J. Strzalka , C. B. Nielsen , S. Barlow , S. R. Marder , I. McCulloch , H. Sirringhaus , Adv. Mater. 2022, 34, 2102988. 10.1002/adma.202102988 34418878

[36] Y. Huang , W. Wen , S. Mukherjee , H. Ade , E. J. Kramer , G. C. Bazan , Adv. Mater. 2014, 26, 4168. 24710682 10.1002/adma.201400497

[37] A. Liu , S. Zhao , S. B. Rim , J. Wu , M. Könemann , P. Erk , P. Peumans , Adv. Mater. 2008, 20, 1065.

[38] H. Chen , R. Zhang , X. Chen , G. Zeng , L. Kobera , S. Abbrent , B. Zhang , W. Chen , G. Xu , J. Oh , S.‐H. Kang , S. Chen , C. Yang , J. Brus , J. Hou , F. Gao , Y. Li , Y. Li , Nat. Energy 2021, 6, 1045.

[39] B. Zhang , F. Yang , S. Chen , H. Chen , G. Zeng , Y. Shen , Y. Li , Y. Li , Adv. Funct. Mater. 2022, 32, 2202011.

[40] W. Xue , Z. Zhang , S. K. So , Y. Song , Z. Wei , W. Ma , H. Yan , ACS Appl. Energy Mater. 2022, 5, 9929.

[41] Y. Cui , H. Yao , J. Zhang , K. Xian , T. Zhang , L. Hong , Y. Wang , Y. Xu , K. Ma , C. An , C. He , Z. Wei , F. Gao , J. Hou , Adv. Mater. 2020, 32, 1908205. 10.1002/adma.201908205 32227399

[42] P. Wei , J. H. Oh , G. Dong , Z. Bao , J. Am. Chem. Soc. 2010, 132, 8852. 20552967 10.1021/ja103173m

[43] Y. Tang , H. Zheng , X. Zhou , Z. Tang , W. Ma , H. Yan , Energy Environ. Sci. 2023, 16, 653.

[44] M. P. Hein , A. A. Zakhidov , B. Lüssem , J. Jankowski , M. L. Tietze , M. K. Riede , K. Leo , Appl. Phys. Lett. 2014, 104, 013507.

[45] J. Belasco , S. K. Mohapatra , Y. Zhang , S. Barlow , S. R. Marder , A. Kahn , Appl. Phys. Lett. 2014, 105, 063301.

[46] M. Nikolka , G. Schweicher , J. Armitage , I. Nasrallah , C. Jellett , Z. Guo , M. Hurhangee , A. Sadhanala , I. McCulloch , C. B. Nielsen , H. Sirringhaus , Adv. Mater. 2018, 30, 1801874. 10.1002/adma.201801874 30022541

[47] W. Xue , Z. Liang , Y. Tang , C. Zhao , L. Yan , W. Ma , H. Yan , Adv. Funct. Mater. 2023, 33, 2304960.

