# Supplementary material for: Eliminating the Imbalanced Mobility Bottlenecks via Reshaping Internal Potential Distribution in Organic Photovoltaics
Source: Adv Sci (Weinh). 2023 Aug 27;10(29):2302880. doi: 10.1002/advs.202302880 (PMC10582413; doi:10.1002/advs.202302880)
Supplement: Supplementary file 1 — Supporting Information [file ADVS-10-2302880-s001.pdf]

ADVANCED SCIENCE
Open Access

Supporting Information

for *Adv. Sci.*, DOI 10.1002/advs.202302880

Eliminating the Imbalanced Mobility Bottlenecks via Reshaping Internal Potential Distribution in Organic Photovoltaics

*Yu Cui, Chao Zhao, João Paulo Araújo Souza, Leandro Benatto, Marlus Koehler, Wei Ma and Han Yan**

Supporting Information

Eliminating the imbalanced mobility bottlenecks via reshaping internal potential distribution in organic photovoltaics

*Yu Cui, Chao Zhao, João Paulo Araújo Souza, Leandro Benatto, Marlus Koehler, Wei Ma and Han Yan**

Y. Cui, Dr. C. Zhao, Prof. W. Ma, Prof. H. Yan

State Key Laboratory for Mechanical Behavior of Materials; Xi'an Jiaotong University; Xi'an 710049, P. R. China

E-mail: mseyanhan@mail.xjtu.edu.cn

J. P. A. Souza, Dr. L. Benatto, Prof. M. Koehler

Department of Physics; Federal University of Paraná; 81531-980 Curitiba, PR, Brazil

Materials. PTQ10, PM6 and BTP-eC9 were purchased from Solarmer Materials Inc. Y6 was purchased from eFlexPV Inc. BCF, N-DMBI, acetonitrile, polystyrene (PS)

and Chloroform (CF) were purchased from Sigma-Aldrich. All chemicals were used as received without further purification.

BHJ device fabrication. BCF·H_2O and N-DMBI were dissolved in acetonitrile solvent with different concentrations (0.1mg/mL, 0.01mg/mL, 0.001mg/mL) and stirring on a hot plate at room temperature. PTQ10:Y6 (20 mg/mL, D/A = 1:1.2) and PM6:BTP-eC9 (16 mg/mL, D/A = 1:1.2) were dissolved in CF solvent and stirring on a hot plate at 50 °C. The cathode p-doped organic solar cell was fabricated in a traditional device structure of ITO/PEDOT:PSS/active layer/PDINO/Ag. The anode n-doped organic solar cell was fabricated in a traditional device structure of ITO/ZnO/active layer/MoO_3/Ag. The blend solutions were spin-coated on corresponding interface layers. Dopant solution was spin-coated on the active layer at 5500 rpm for 30 s. For cathode p-doped devices, a thin PDINO layer (2 mg/mL in methanol, 3300 rpm for 30 s) was spin coated on the active layer. For anode n-doped devices, a 10 nm MoO_3 layer was deposited on the active layer. Finally, 100 nm Ag was deposited at a vacuum level of < 4×10^{-5} Pa. Typical devices area (0.04 cm^2) was defined by a metal mask with aligned aperture.

Double-BHJ-layer device fabrication. The bilayer devices were fabricated with an conventional device configuration of ITO/PEDOT:PSS/Active Layer/PDINO/Al. The donor and acceptor (D:A=1:1.2, 18 mg/mL) were dissolved in CF. The double-BHJ-layer was fabricated by the floating-film-transfer method as used in our previous work. The top BHJ layer was spin-coated on PEDOT:PSS. The dopant blocking layer, PS (molecule weight 19.8 M) in ethyl acetate solution (5mg/mL) was spin-coated on upper BHJ layer at 4500 rpm for 30 s. The top layer was floated in water. The bottom BHJ layer was also spin-coated on PEDOT:PSS. Then we used the BHJ-coated PEDOT:PSS/ITO substrate to stick on top of the floated film. After that, the samples were placed in a vacuum overnight at a vacuum level of $<1 \times 10^{-4}$ Pa and spin-coated BCF·H_2O solution at 3000rpm for 25s. The complex cathode structure was fabricated as the same procedure as the BHJ device.

Semitransparent device fabrication. PM6:BTP-eC9 (16 mg/ml, D/A = 1:3) were dissolved in CF solvent and stirring on a hot plate at 50 °C. After that, the blend solutions were spin-coated on ZnO to obtain similar film thickness of 75 nm ± 2 nm. Then a 10 nm MoO_3 layer was deposited on the active layer. For the semi-transparent organic solar cell, ultrathin Ag and 35 nm MoO3 replaced the 100 nm Ag. The 15 nm Ag was deposited at a rate of 2 Å·s^{-1} and the 35 nm MoO_3 was deposited at a rate of 0.1 Å·s^{-1}.

DDG simulation. We adopt the classic drift-diffusion-generation (DDG) model as Koster. et al proposed, with doping being taken into account. The Poisson equation for the electric field strength E in the presence of dopants is:

$$\frac{dE}{dx}=\frac{e}{\varepsilon\varepsilon_o}(p-n+N_n-N_p)$$

where N_n and N_p are n- and p-type concentrations of ionized dopants, respectively, e is the elementary charge, p and n are the concentrations of free holes and electrons, ε is the relative dielectric constant, ε_o is the vacuum permittivity, and x is the spatial coordinate. In organic semiconductor, the concentration of ionized uncompensated dopants can reach 10^{24} m^{-3}. Since ion current is neglective, the electron current, hole current, and continuity equations are the same as the non-doping model at quasi-steady state:

$$J_n = e\mu_n(n\frac{dV}{dx} - V_t\frac{dn}{dx})$$

$$J_p = e\mu_p(p\frac{dV}{dx} + V_t\frac{dp}{dx})$$

$$\frac{dn}{dt} = PG - (1-P)R + \frac{dJ_n}{dx}$$

$$\frac{dp}{dt} = PG - (1-P)R - \frac{dJ_p}{dx}$$

where $V_t = \frac{k_B T}{e}$ is the thermal voltage, μ_h and μ_p are the electron and hole mobility, and V is the electric potential. The charge-generation and recombination processes are described by the current continuity equations for electrons and holes. G is the generation rate of bound electron-hole pairs, P(E) is the probability of bound

electron-hole pair dissociation. The current densities of electrons J_n and holes J_p are presented as a sum of drift and diffusion current densities. The total current density through the active layer is a sum of the electron and hole current densities $J = J_n + J_p$, as shown in Supplementary Figure S1.

DFT and TD-DFT calculation. We apply density functional theory (DFT) and time-dependent density functional theory (TD-DFT) to study the interaction among an oligomer of PTQ10 with two repeating units, Y6, water and BCF. All DFT and TD-DFT calculations in this work were performed using the Gaussian 16 package. [1]

As a first step, the gas-phase ground state geometry of the molecules were determined using the ωB97XD[2] functional, which is capable of capturing short- and long-range interactions, along with the base set 6-31G (d,p).[3, 4] We then used the same functional and base set to perform all the remaining DFT calculations.

In order to evaluate the intensity of the interaction between the BCF(OH_2) adduct with the dimer of PTQ10 or with the Y6, we optimized the complex formed by the three molecules. We started with a initial configuration were the water molecule is located between the BCF and the polymer or the acceptor. In the case of Y6, two different initial configurations were tested, one with the BCF located near the Y6 edge an another one with the BCF positioned near the central groups of the acceptor. After running the optimization of the complex, a vibrational analysis was performed for the resulting configuration of the system. The Gibss free energy of the complex (ΔG_c) was then estimated directly from this DFT calculation. We use the same procedure to estimate the Gibss free energy of the isolated molecules (ΔG). The variation of the Gibss free energy due to the formation of the BCF(OH_2)/oligomer or BCF(OH_2)/Y6 complexes was estimated by the difference between ΔG_c and the sum of the ΔG's of the isolated molecules.

GIWAXS characterization. GIWAXS measurements were performed at beamline 7.3.3[5] at the Advanced Light Source. Samples were prepared on Si substrates using identical blend solutions as those used in devices. The 10 keV X-ray beam was

incident at a grazing angle of 0.11°-0.15°, selected to maximize the scattering intensity from the samples. The scattered x-rays were detected using a Dectris Pilatus 2M photon counting detector.

Calculating the SCLC mobility. The SCLC mobility (μ) was measured with the hole-only device structure of ITO/PEDOT:PSS/active layer/MoO_3/Al and electron-only device structure of ITO/ZnO/active layer/PFN-Br/Al. The values of SCLC mobility were obtained by fitting the current density-voltage curves according to

$$J=\frac{9\varepsilon_0\varepsilon_r\mu V^2}{8d^3}$$

where ε_0 is the permittivity of vacuum, ε_r is the relative permittivity of the active layer and it is assumed to be 3.5 here.

Flory-Huggins interaction parameter. The polymer solvent interaction parameter, which reflects the change in the interaction energy during the mixing of polymer molecules with the solvent, is expressed as χ. From the derivation of the thermodynamic theory of polymer solution, it is known that the value of polymer solvent interaction parameter χ can be used as a semi-quantitative criterion for the superiority of solvent. If χ is greater than 0.5, the polymer generally cannot be dissolved; if χ is less than 0.5, the polymer can be dissolved, and the smaller it is, the better the solvency ability of the solvent. Therefore, the value of χ can be used as a basis to determine whether the polymer and solvent system are mutually soluble. We first build the polymer 3D model, and then import it into HSPiP software to calculate the polymer-solvent interaction parameters.

Other measurements. TM-AFM images were scanned by Bruker INNOVA. The J-V curves were performed in the N_2-filled glovebox under AM 1.5G (100 mW cm^{-2}) using an AAA solar simulator (SS-F5-3A, Enli Technology Co., Ltd.) calibrated with a standard photovoltaic cell equipped with KG5 filter. The EQE curves were

measured by Solar Cell Spectral Response Measurement System QE-R3018 (Enli Technology Co., Ltd.) with calibrated light intensity by a standard Si photovoltaic cell. The transmittance was obtained on a Shimadzu UV-3600 Plus Spectrophotometer. For the semitransparent devices, the reference is air. The EQE spectrum was obtained by using the corrected Si standard detector (S1337-1010Br). ESR spectra were tested on Bruker Biospin A300-9.5/12. TOF-SIMS were tested on ION-TOF M6.

AVT. The AVT value was calculated according to the average value of transmittance of semitransparent devices based on photonic response of the human eye. The wavelength range is usually adopted by 380-760nm, and the specific calculation formula is

$$AVT=\frac{\int \mathrm{T}(\lambda)\mathrm{V}(\lambda)\mathrm{S}(\lambda)\mathrm{d}(\lambda)}{\int \mathrm{P}(\lambda)\mathrm{S}(\lambda)\mathrm{d}(\lambda)}$$

where λ is the wavelength, T is the transmission, V is the normalized photopic spectral

response of the eye, and S is the solar photon flux (AM1.5G).

Color coordinates. The color coordinates (x, y, z) of semi-transparent devices were calculated according to the transmission spectra based on chromaticity diagram of the CIE 1931xy. The color coordinates were calculated by the formulas

$$\begin{cases} X=\int \varphi(\lambda) * \bar{\mathrm{x}}(\lambda) * \mathrm{d}(\lambda) \\ Y=\int \varphi(\lambda) * \bar{\mathrm{y}}(\lambda) * \mathrm{d}(\lambda) \\ Z=\int \varphi(\lambda) * \bar{\mathrm{z}}(\lambda) * \mathrm{d}(\lambda) \end{cases}$$

$$\begin{cases} \mathbf{x}=\frac{\mathrm{X}}{\mathrm{X+Y+Z}} \\ \mathbf{y}=\frac{\mathrm{Y}}{\mathrm{X+Y+Z}} \\ \mathbf{x}=\frac{\mathrm{Z}}{\mathrm{X+Y+Z}} \end{cases}$$

where *X*, *Y*, *Z* are tristimulus values, $\varphi(\lambda)$ is the spectral power distribution (SPD) of the transmission spectra of semi-transparent devices and $\bar{x}(\lambda)$, $\bar{y}(\lambda)$, $\bar{z}(\lambda)$ are color-matching functions.

CCT and CRI. The CIE 1960 UCS diagram is a graph of u and v values. The iso-temperature is a constant color temperature line for a blackbody radiator and can be drawn on this 1960 UCS diagram. The CCT of the test source can be obtained by projecting the calculated chromaticity coordinate values (u_t, v_t) onto this UCS diagram. the temperature corresponding to the blackbody radiator closest to the chromaticity coordinate (u_t, v_t) is determined as the CCT.

The definition of color includes chromaticity (u, v) and luminous intensity L (the "brightness" of the light source and the "luminosity" of the physical object). When illuminated with a reference or transmitted source, a color sample (i) will exhibit color differences consisting of chromaticity differences (Δu_i^* and Δv_i^*) and luminance differences (ΔL_i^*). Since the shape of T(λ) determines the extent to which a transmitted source can maintain the color rendering of AM1.5G, the geometric distance between the point of the transmitted source and the point of the reference AM1.5G in the chromaticity coordinate system accounts for the chromaticity difference. There were eight standard test color samples used as the basis for these chromaticity and luminance differences, and these chromaticity and luminance differences are averaged to calculate the CRI:

$$\mathrm{CRI}=\frac{1}{8}\sum_{i=1}^{8}[100-4.6\cdot\sqrt{(\Delta u_i^*)^2+(\Delta v_i^*)^2+(\Delta L_i^*)^2}]$$

The above calculation done by IES TM-30-18 Advanced Calculation Tool.

The IES TM-30-18 method. R_f is a measure of average color fidelity and is calculated by determining the difference in CAM02-UCS coordinates for each CES at the test and reference light sources and then determining the arithmetic mean of these color differences. This average should be scaled by a factor of 6.73 and subtracted from 100:

$$R_f' = 100 - 6.73\ [\frac{1}{99}\sum_{i=1}^{99}(\Delta E_{Jab,i})]$$

Then, the scale should be adjusted so that the minimum R_f value is 0 to avoid generating negative numbers. Rescaling to the final R_f value should be accomplished using the following method:

$$R_f = 10\ln\left[\exp(R_f' / 10) + 1\right]$$

The fidelity value for each of the 99 CES may be calculated using the same method as for R_f.

To calculate the remaining specified measures, the 99 CES are divided in 6 groups. The boundaries are established by dividing the $a' - b'$ plane of CAM02-UCS into 16 sections following a radical pattern, with each encompassing 22.5°.

R_g is a measure of the area spanned by the average (a', b') coordinates of the CES in each hue-angle bin, $(a'_{test,j}, b'_{test,j})$ and $(a'_{ref,j}, b'_{ref,j})$. The J' coordinate is discarded, so that the $(a'_{test,j}, b'_{test,j})$ and $(a'_{ref,j}, b'_{ref,j})$ coordinates each form a polygon. Rg is calculated as 100 times the ratio of the area of the two polygons (A_t and A_r, respectively):

$$R_g = 100 \times \frac{A_t}{A_r}$$

The above calculation done by IES TM-30-18 Advanced Calculation Tool.

Supplementary Figures

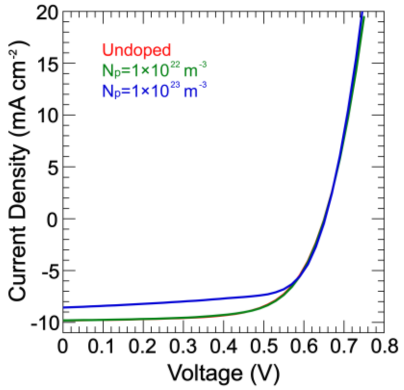

Figure S1. Simulated JV curves, with different doping levels. The device parameters are: 100 nm thick; $\mu_e = \mu_h = 1\times10^{-4}$ cm^2/Vs; $V_{bi} = 0.75$V; contact carrier density: 2×10^{17} cm^{-3}; geminate recombination rate: 10^4 s^{-1}.

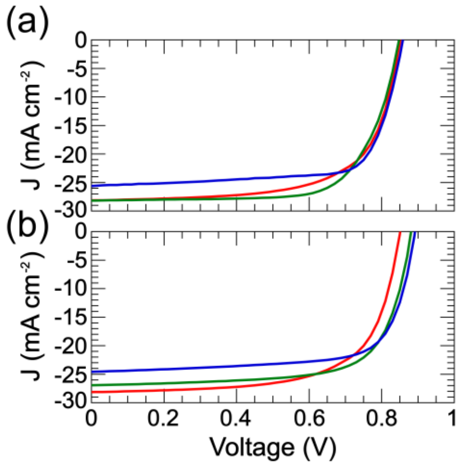

Figure S2. Simulated JV characteristics of (a) *p*-doping at cathode and (b) at anode. The hole mobility is $\mu_h = 1\times10^{-4}$ cm^2/Vs, electron mobility is $\mu_e = 1\times10^{-3}$ cm^2/Vs with different doping depth. Doping density is 2×10^{17} cm^{-3}. from red line to black line, the doping depth increases at a step of 10nm. The hole-density-dependent mobility follows Gaussian disordering theory: $\mu_p = \mu_{o,p} (1 + \alpha (n_p/N_{site})^{0.50}$, where n_p is hole density, $\mu_{o,p}$ is limiting hole mobility at low density (1×10^4 cm^2/Vs), α is hole-density coefficient, and N_{site} is transport site density. For parameter values, see caption of Table S1.

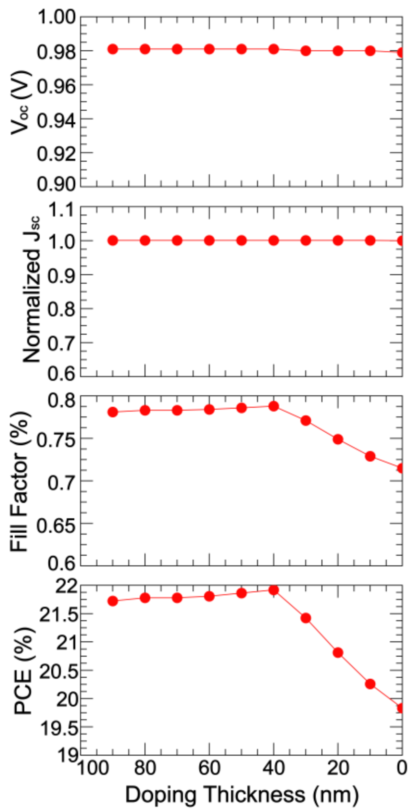

Figure S3. Device performance of *p*-doped anode with different doping depth, where both electrodes are selective contacts, i.e., $J_e = 0$ at anode and $J_h = 0$ at cathode. The *p*-doping density is 2×10^{17} cm^{-3}, doping depth is 60 nm.$\mu_e = 1\times10^{-3}$ cm^2/Vs, $\mu_h = 1\times10^{-4}$ cm^2/Vs. $V_{bi} = 0.9$V, $k_f = 10^3$ s^{-1}.

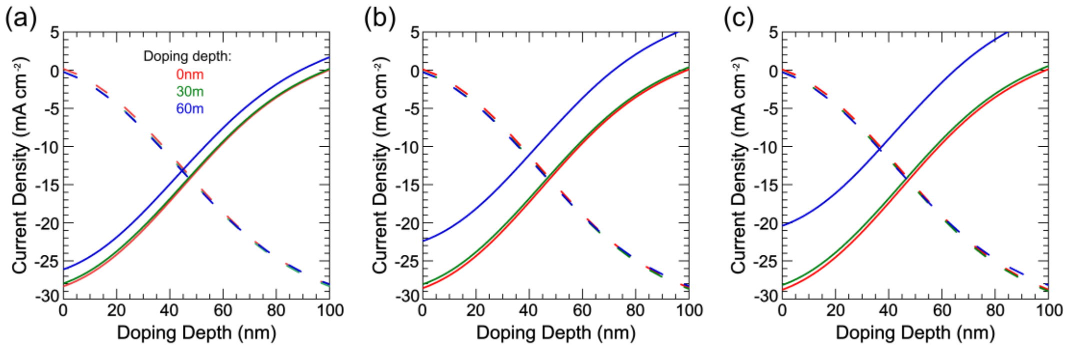

Figure S4. Simulated electron current and hole current profile. *p*-doping at cathode: (a) $\mu_h = 1\times10^{-4}$ cm^2/Vs; (b) $\mu_h = 3\times10^{-4}$ cm^2/Vs; (c) $\mu_h = 1\times10^{-3}$ cm^2/Vs. The electron mobility is fixed at $\mu_e = 1\times10^{-3}$ cm^2/Vs. Doping density is 2×10^{17} cm^{-3}. Electron current: solid lines; Hole current: dashed lines.

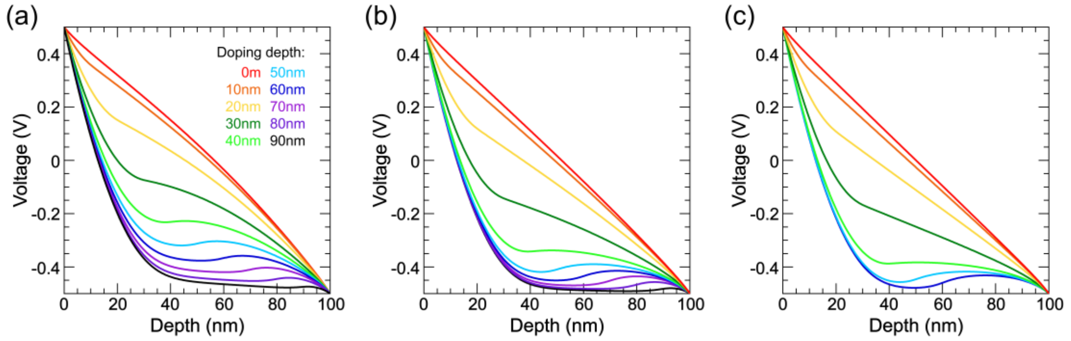

Figure S5. Simulated electric potential profile at J_{SC} condition. *p*-doping at cathode: (a) μ_h = 1×10^{-4} cm^2/Vs; (b) μ_h = 3×10^{-4} cm^2/Vs; (c) μ_h = 1×10^{-3} cm^2/Vs. The electron mobility is fixed at μ_e = 1×10^{-3} cm^2/Vs. Doping density is 2 ×10^{17} cm^{-3}. from red line to black line, the doping depth increases at a step of 10nm.

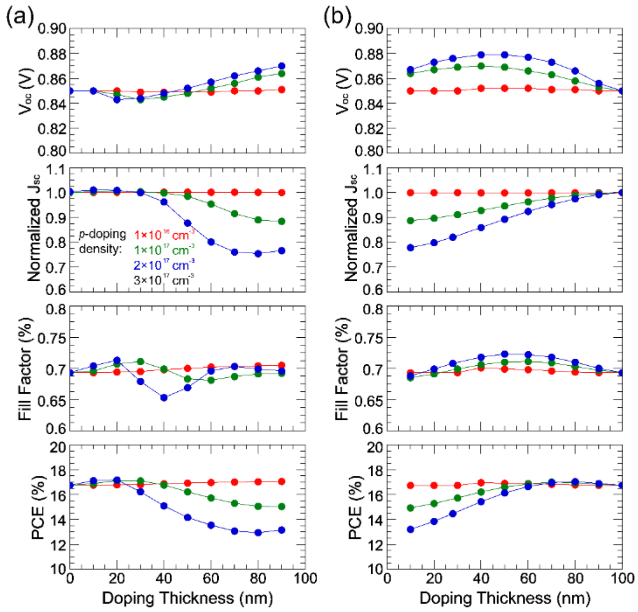

Figure S6. Simulated device performance of *p*-doping at (a) cathode and (b) anode with different doping depth. μ_e = 1×10^{-3} cm^2/Vs , μ_h = 3×10^{-4} cm^2/Vs . The *p*-doping density is 1×10^{16} cm^{-3} (red lines), 1×10^{17} cm^{-3} (green lines), 2×10^{17} cm^{-3} (blue lines), 3×10^{17} cm^{-3} (black lines). V_{bi} = 0.9V, k_f = 10^3 s^{-1}.

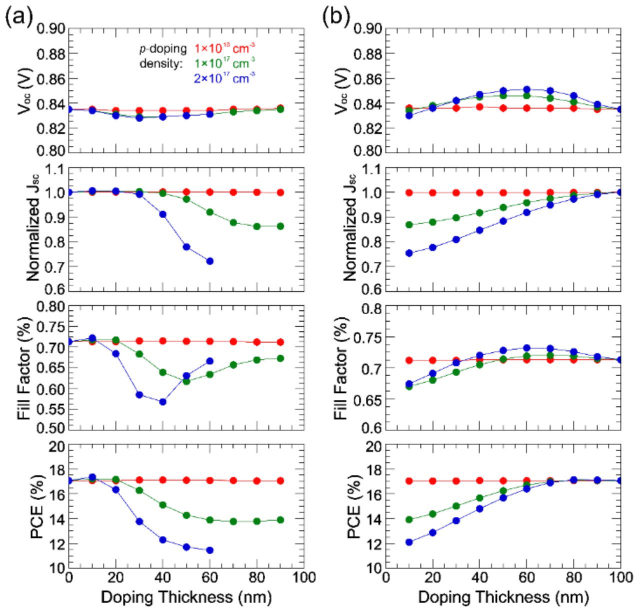

Figure S7. Simulated device performance of *p*-doping at (a) cathode and (b) anode with different doping depth. $\mu_e = 1\times10^{-3}$ cm^2/Vs, $\mu_h = 1\times10^{-3}$ cm^2/Vs . The *p*-doping density is 1×10^{16} cm^{-3} (red lines), 1×10^{17} cm^{-3} (green lines), 2×10^{17} cm^{-3} (blue lines), 3×10^{17} cm^{-3} (black lines). V_{bi} = 0.9V, $k_f = 10^3$ s^{-1}.

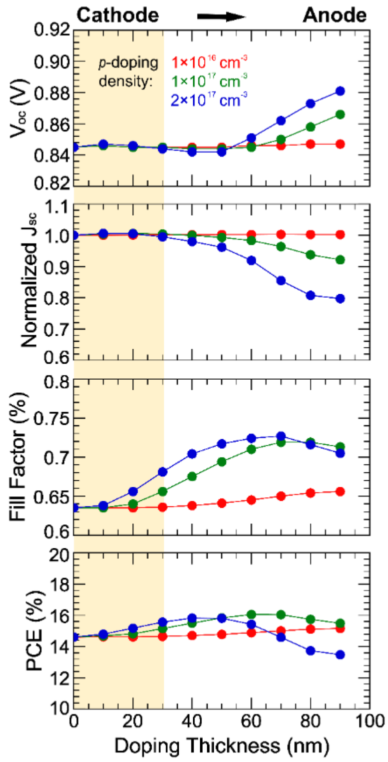

Figure S8. Simulated device performance of *p*-doping at cathode with different doping depth. $\mu_e = 1\times10^{-3}$ cm^2/Vs , $\mu_h = 1\times10^{-4}$ cm^2/Vs . The *p*-doping density is 1×10^{16} cm^{-3} (red lines), 1×10^{17} cm^{-3} (green lines), 2×10^{17} cm^{-3} (blue lines). V_{bi} = 0.9V, $k_f = 10^4$ s^{-1}.

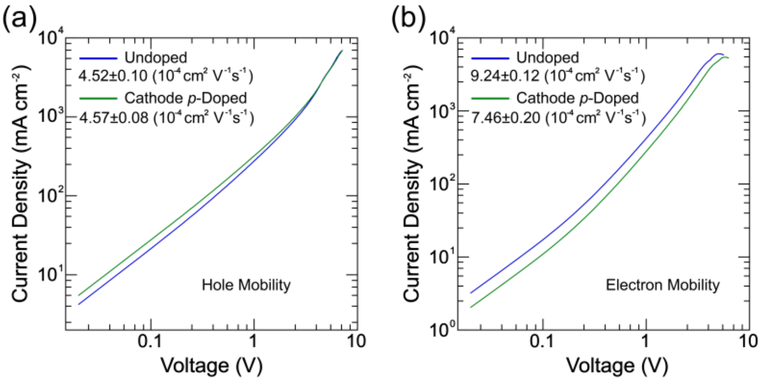

Figure S9. Hole-only and electron-only charge transport curves of the control and cathode p-doped PTQ10:Y6 devices

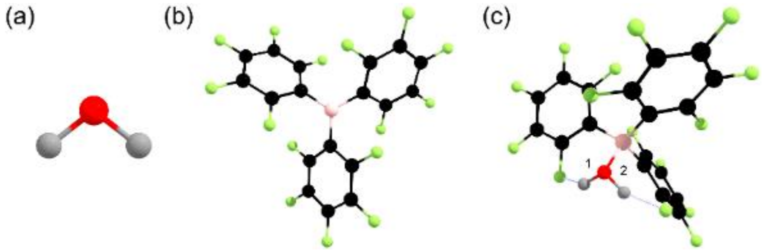

Figure S10. Interaction of the water molecule with the p-dopant BCF.

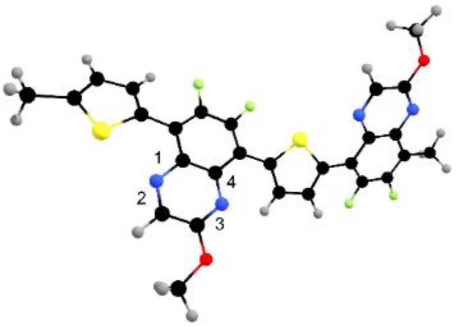

Figure S11. Isolated polymer PTQ10.

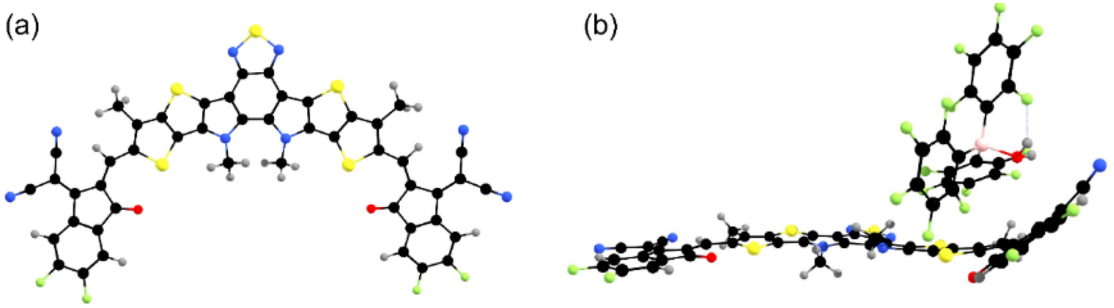

Figure S12. (a) Y6 molecule and (b) Interaction of Y6 molecules and fluorine in hydrated BCF.

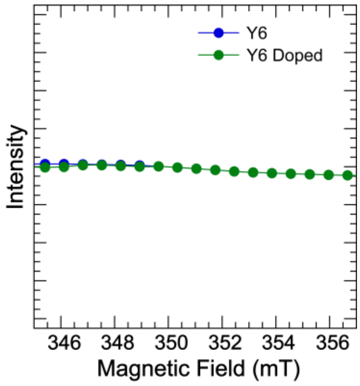

Figure S13. EPR spectra of Y6 and BCF doped Y6 in film.

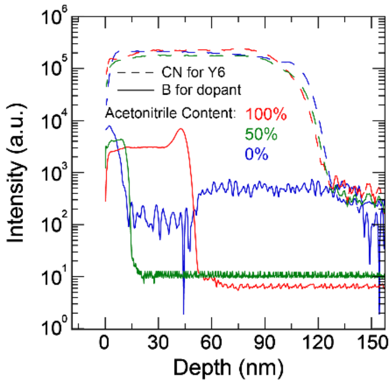

Figure S14. TOF-SIMS depth profile of PTQ10:Y6 films under three doping conditions (Acetonitrile content of dopant solvent: 100%, 50%, 0%).

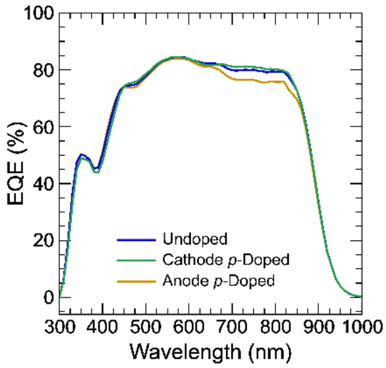

Figure S15. EQE curves of undoped, cathode p-doped and anode p-doped PTQ10:Y6 BHJ device.

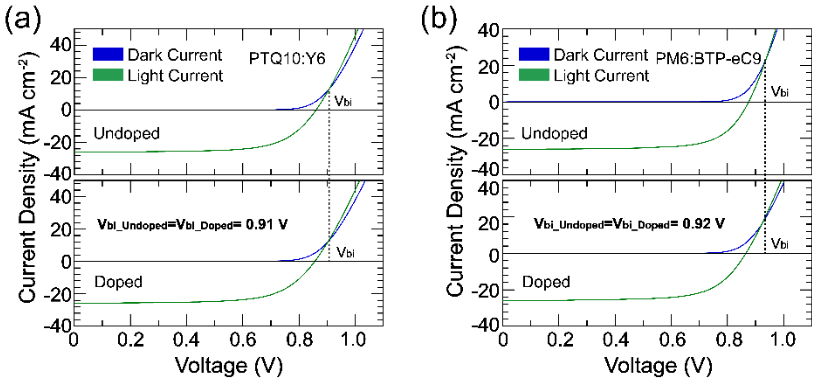

Figure S16. Curves of device light/dark currents density before and after doping: (a) cathode *p*-doped PTQ10:Y6, (b) anode *n*-doped PM6:BTP-eC9.

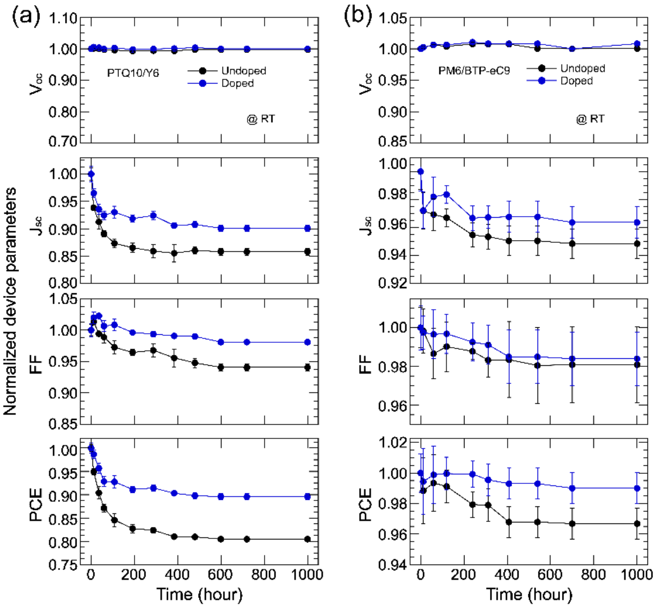

Figure S17. Evolution of photovoltaic parameters under dark aging condition for up to 1000 h storage of: (a) cathode *p*-doped and undoped devices, (b) anode *n*-doped and undoped devices.

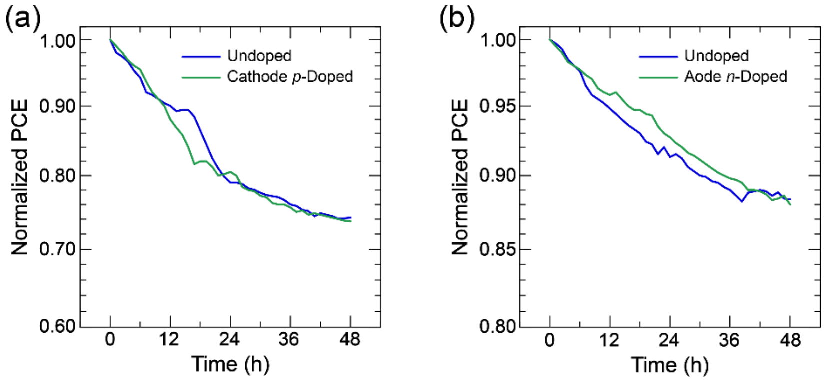

Figure S18. MPP stability test of unencapsulated OSCs based on (a) undoped and

cathode *p*-doped PTQ10:Y6, (b) undoped and anode *n*-doped PM6:BTP-eC9 respectively, stored in N_2 atmosphere under the illumination of AM 1.5G 100 mW cm^{-2}.

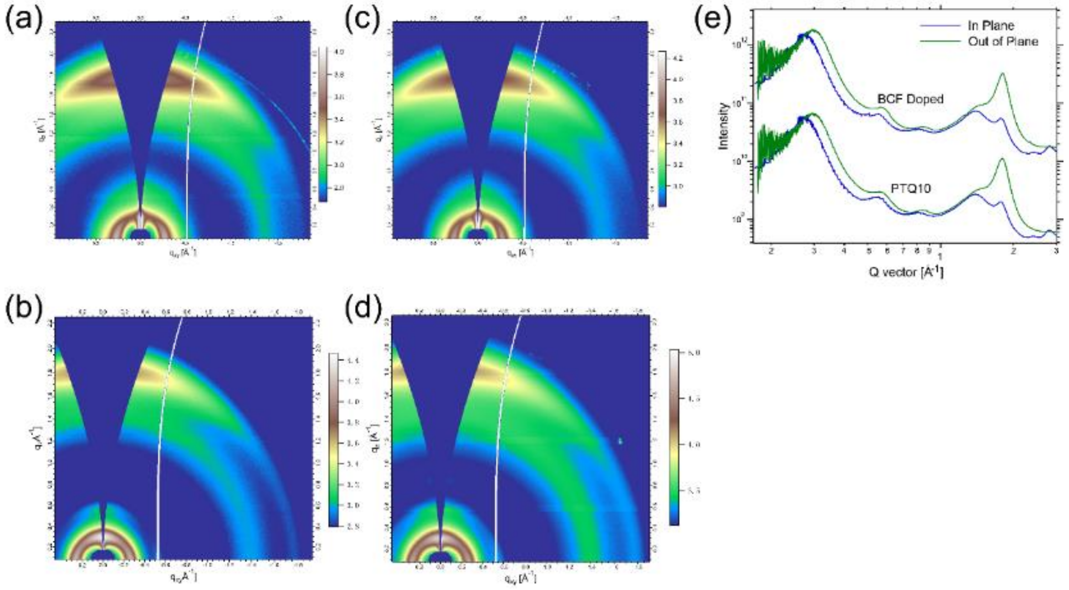

Figure S19. 2D GIXD patterns of (a) BCF doped and (c) undoped PTQ10:Y6 blended films, and (b) BCF doped and (d) undoped PTQ10 films. The in-plane (IP) and out-of-plane (OOP) line cut profiles data based on BCF doped and undoped PTQ10 films.

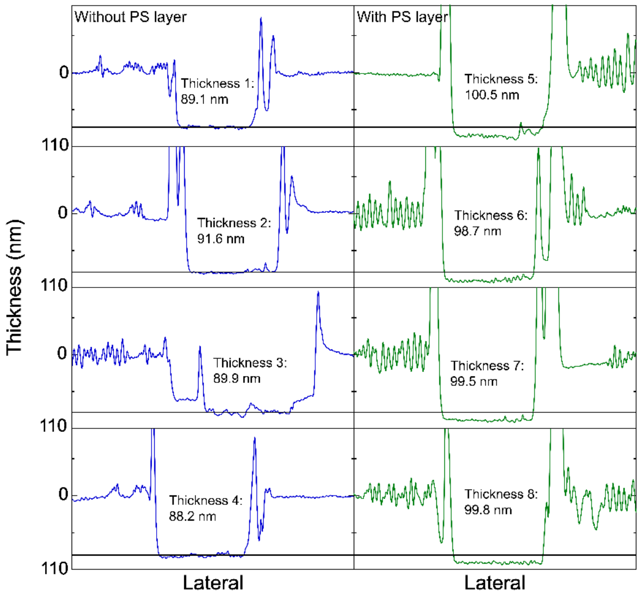

Figure S20. Thickness of thin films without/with PS layer measured by the step profiler.

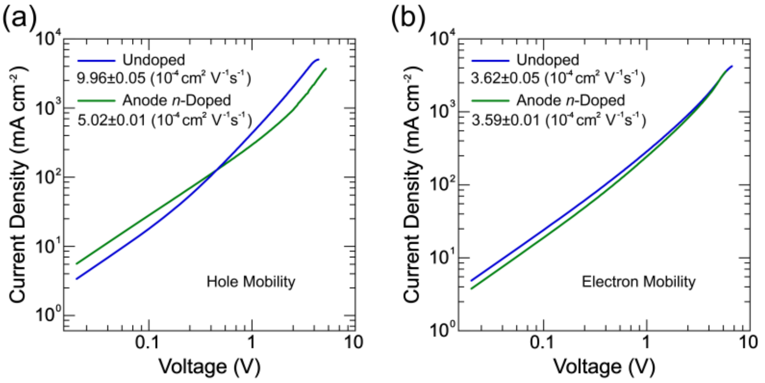

Figure S21. Hole-only and electron-only charge transport curves of the control and anode n-doped PM6:BTP-eC9 device.

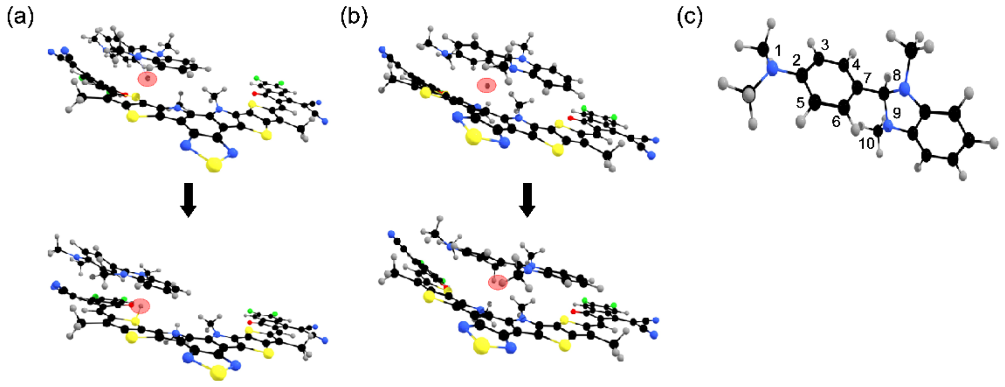

Figure S22. (a) H atom, which came out of the n-dopant, interacting with the sulfur atom of the BTP-eC9 and (b) H atom interacting with the carbon atom of the N-DMBI itself. (c) N-DMBI molecule.

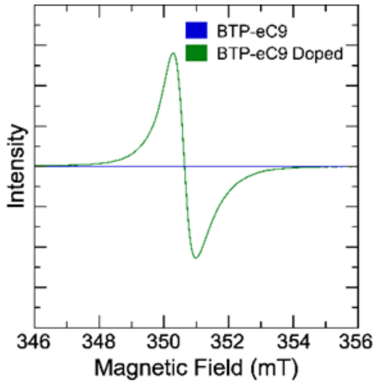

Figure S23. ESR spectra of undoped and doped BTP-eC9.

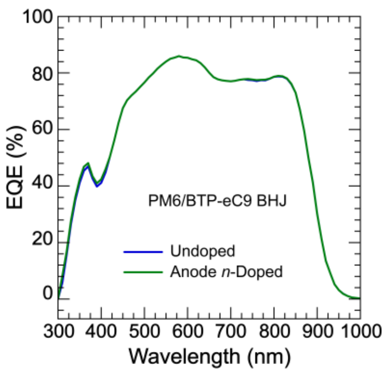

Figure S24. EQE curves of undoped and anode n-doped PM6:BTP-eC9 BHJ device.

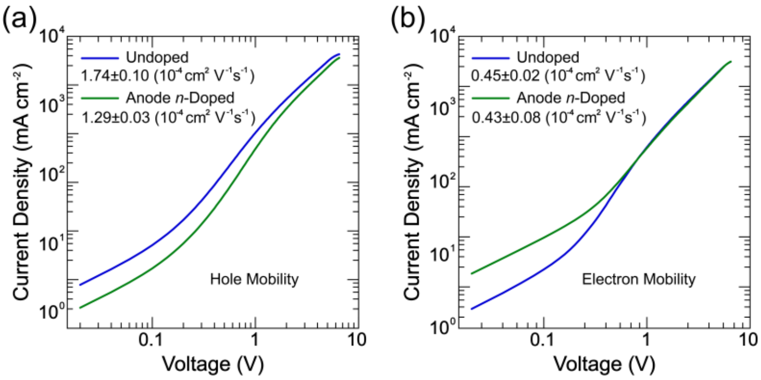

Figure S25. Hole-only and electron-only charge transport curves of the control and anode n-doped semitransparent devices.

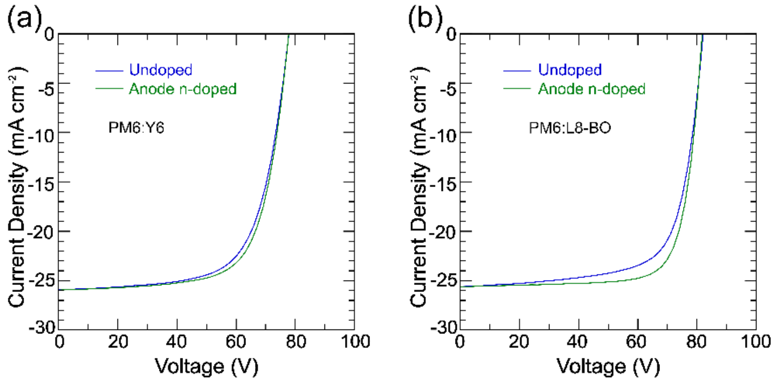

Figure S26. J-V curves of the undoped and n-doped devices based on different Y-series acceptors with a device area of 4 mm^2.

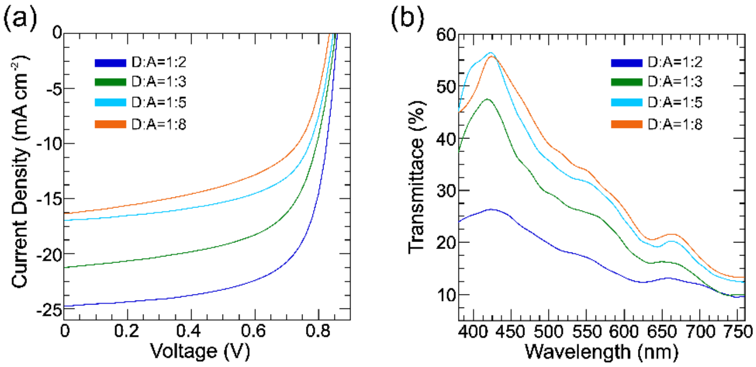

Figure S27. The J-V curve and transmittance spectra of undoped semitransparent devices with various PM6:BTP-eC9 weight ratios (1:2, 1:3, 1:5 and 1:8).

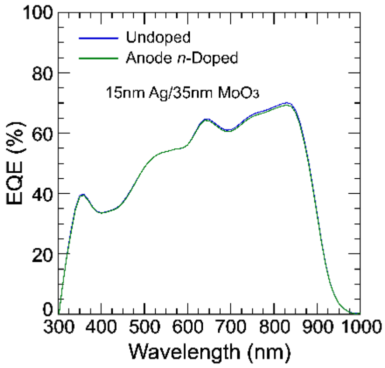

Figure S28. EQE curves of undoped and anode n-doped PM6:BTP-eC9 semitransparent device.

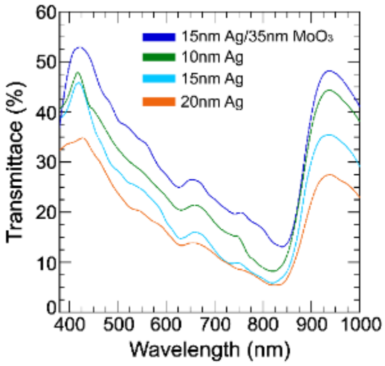

Figure S29. The transmittance spectra of undoped PM6:BTP-eC9 semitransparent devices with various Ag electrode thickness (10nm, 15nm, 20nm and 15nmAg/35nm MoO_3).

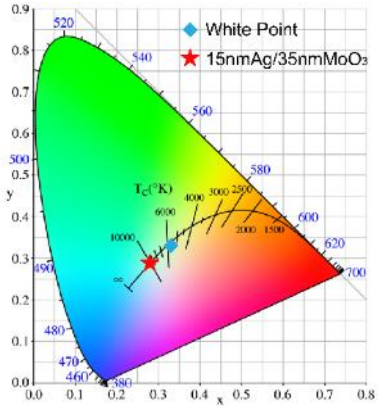

Figure S30. Color coordinates of white point and optimal semitransparent devices.

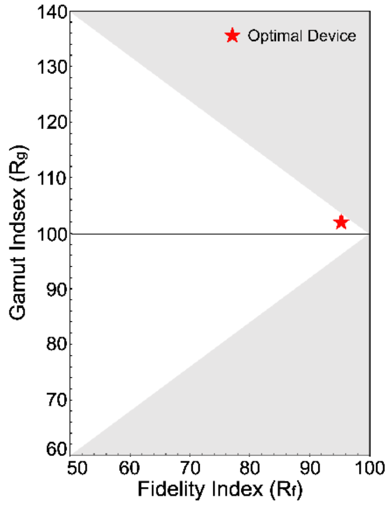

Figure S31. Gamut Index vs. Fidelity Index. The range in possible R_g values increases as R_f decreases. The gray shaded area indicates the approximate region of combinations that are not possible for nominally white light sources.

Supplementary Tables

Table S1. Simulated device parameters of cathode p-doping and anode p-doping with different doping depth. Doping density is 2×10^{23} m^{-3}. The hole mobility is $\mu_h = 1\times10^{-8}$ m^2 V^{-1}s^{-1}, electron mobility is $\mu_e = 1\times10^{-7}$ m^2 V^{-1}s^{-1} with different doping depth.

**Cathode p-doping depth (nm)**	**V_{OC} (V)**	**J_{SC} (mA cm^{-2})**	**Fill Factor (%)**	**PCE (%)**
0	0.849	28.1	64.8	15.5
30	0.847	28.1	69.2	17.1
60	0.853	26.4	74.7	16.8
Anode p-doping depth (nm)	V_{OC} (V)	J_{SC} (mA cm^{-2})	FF (%)	PCE (%)
0	0.849	28.1	64.8	15.5
30	0.879	27.0	69.7	16.5
60	0.890	24.9	72.0	15.9

Table S2. Simulated device parameters of cathode p-doping with different μ_h/μ_e in the case of 30nm doping depth.

**Condition**	**V_{OC} (V)**	**J_{SC} (mA cm^{-2})**	**FF (%)**	**PCE (%)**
Control	0.849	28.1	64.8	15.5
μ_h/μ_e=1/10	0.847	28.1	69.2	17.1
μ_h/μ_e=3/10	0.844	28.3	67.9	16.2
μ_h/μ_e=10/10	0.828	28.4	58.5	13.8

Table S3. Variation in the bond length of the interaction between PTQ10, H_2O and p-dopant BCF.

**Points**	**Before interaction – Figure S11 (Å)**	**After interaction – Figure 3b (Å)**
1	1.350	1.350
2	1.306	1.306
3	1.304	1.310
4	1.350	1.360

Table S4. Flory-Huggins interaction parameters between donor/acceptor and different solvents.

	PTQ10	Y6	PM6	BTP-eC9
Acetonitrile	$\chi_{ACN/PTQ10}$=0.88	$\chi_{ACN/PTQ10}$=0.94	$\chi_{ACN/PTQ10}$=0.73	$\chi_{ACN/PTQ10}$=0.94
Ethanol	$\chi_{ACN/PTQ10}$=0.96	$\chi_{ACN/PTQ10}$=0.95	$\chi_{ACN/PTQ10}$=0.90	$\chi_{ACN/PTQ10}$=0.95

Table S5. Device parameters of cathode p-doped PTQ10:Y6 devices with different dopant concentration. [a]

**Condition**	**V_{OC} (V)**	**J_{SC} (mA cm^{-2})**	**FF (%)**	**PCE (%)**
Control	0.878 (0.877±0.001)	26.3 (26.0±0.1)	67.5 (67.2±0.3)	15.4 (15.3±0.1)
BCF 0.001mg/ml	0.881 (0.878±0.002)	26.2 (26.1±0.2)	69.9 (69.3±0.2)	15.8 (15.7±0.1)
BCF 0.01mg/ml	0.878 (0.876±0.003)	26.3 (26.1±0.1)	70.9 (70.3±0.2)	16.2 (16.1±0.1)
BCF 0.1mg/ml	0.882 (0.878±0.002)	26.2 (26.1±0.1)	70.6 (70.1±0.3)	16.1 (16.0±0.1)

[a] The parameters are obtained from 10 independent devices.

Table S6. The π-π stacking peak positions and coherence lengths from GIWAXS patterns for undoped and cathode p-doped PTQ10 film and PTQ10:Y6 blended film. Position and FWHM are available through multi-peak fitting and *d*-spacing, CLs can be calculated by Scherrer Equation.

PTQ10	(100)	location	d-spacing	FWHM	CLs
	Control	0.274	22.91	0.083	68.03
	Doped	0.274	22.91	0.082	68.93
	(010)	location	d-spacing	FWHM	CLs
	Control	0.274	22.91	0.083	68.03
	Doped	0.274	22.91	0.081	69.78
PTQ10:Y6	(110)	location	d-spacing	FWHM	CLs
	Control	0.280	22.44	0.104	54.37
	Doped	0.279	22.52	0.103	54.90
	(010)	location	d-spacing	FWHM	CLs
	Control	1.753	3.58	0.216	26.18
	Doped	1.750	3.59	0.217	26.06
	(11-1)	location	d-spacing	FWHM	CLs

Control	0.431	14.58	0.062	91.20
Doped	0.430	14.61	0.061	92.70

Table S7. Double-BHJ devices photovoltaic parameters of OSCs under an illumination of air mass 1.5 global (AM 1.5G), 100 mW cm^{-2}. [a]

Condition		V_{OC} (V)	J_{SC} (mA cm^{-2})	FF (%)	PCE (%)
Doped/Undoped 20nm/65nm	Undoped	0.835 (0.834±0.001)	22.4 (22.2±0.2)	49.8 (48.8±0.6)	9.1 (9.0±0.1)
	Doped	0.836 (0.833±0.002)	22.9 (22.5±0.4)	52.1 (51.5±0.4)	9.6 (9.3±0.2)
Doped/Undoped 30nm/55nm	Undoped	0.836 (0.833±0.001)	22.7 (22.4±0.2)	49.0 (48.3±0.5)	9.0 (8.9±0.1)
	Doped	0.840 (0.838±0.002)	22.7 (22.2±0.4)	53.8 (53.3±0.4)	10.4 (10.0±0.2)
Doped/Undoped 42nm/42nm	Undoped	0.840 (0.838±0.001)	22.6 (22.5±0.1)	49.5 (48.7±0.6)	9.2 (9.1±0.1)
	Doped	0.840 (0.838±0.002)	22.6 (22.0±0.4)	54.1 (53.9±0.2)	10.5 (10.2±0.2)
Doped/Undoped 55nm/30nm	Undoped	0.838 (0.835±0.002)	22.8 (22.2±0.5)	49.2 (48.6±0.5)	9.1 (8.9±0.1)
	Doped	0.838 (0.836±0.001)	22.8 (22.5±0.3)	53.8 (53.6±0.2)	10.3 (10.2±0.1)

[a] The parameters are obtained from 5 independent devices.

Table S8. Bond length variation of n-dopant N-DMBI during interaction with molecule BT*P*-eC9.

Points	Before interaction (Å)	After interaction (Å)
1	1.38	1.39
2	1.40	1.46
3	1.38	1.35
4	1.38	1.44
5	1.41	1.35
6	1.38	1.50
7	1.39	1.52
8	1.50	1.37
9	1.46	1.39
10	1.46	1.40

Table S9. Charge transfer between the BT*P*-eC9 molecule and n-dopant N-DMBI.

	**Charge transfer (*e*)**
A_1	0.458
A_2	0.940

Table S10. Photovoltaic parameters of anode n-doped PM6:BTP-eC9 devices under an illumination of air mass 1.5 global (AM 1.5G), 100 mW cm^{-2}.[a]

**Condition**	**V_{OC} (V)**	**J_{SC} (mA cm^{-2})**	**FF (%)**	**PCE (%)**
Control	0.835 (0.832±0.002)	26.99 (26.91±0.06)	72.72 (71.29±0.91)	16.91 (16.81±0.12)
N-DMBI 0.1mg/mL	0.835 (0.833±0.001)	26.97 (26.74±0.22)	75.69 (75.08±0.35)	17.69 (17.54±0.32)
N-DMBI 0.01mg/mL	0.836 (0.835±0.001)	26.96 (26.68±0.20)	76.67 (76.39±0.25)	18.03 (17.88±0.10)

[a] The parameters are obtained from 10 independent devices.

Table S11. Photovoltaic parameters of undoped and anode *p*-doped devices based on different Y-series acceptors with a device area of 4 mm^2. [a)] (0.01% mg/ml N-DMBI acetonitrile solution)

Condition	V_{OC} (V)	J_{SC} (mA cm^{-2})	FF (%)	PCE (%)
PM6:Y6	0.855 (0.854±0.001)	26.0 (25.8±0.1)	69.3 (69.0±0.3)	15.4 (15.3±0.1)
Anode *n*-doped	0.855 (0.854±0.002)	25.9 (25.8±0.1)	72.7 (71.6±0.6)	16.1 (15.8±0.3)
PM6:L8-BO	0.898 (0.896±0.001)	25.4 (25.0±0.2)	73.1 (73.0±0.1)	16.6 (16.5±0.1)
Anode *n*-doped	0.898 (0.896±0.001)	25.3 (25.2±0.1)	76.9 (76.4±0.5)	17.5 (17.4±0.1)

a) The average PCEs are obtained from 10 devices.

Table S12. Photovoltaic parameters of semitransparent devices with different donor/acceptor ratios for 15 nm Ag electrode under an illumination of air mass 1.5 global (AM 1.5G), 100 mW cm^{-2}.[a]

**Condition**	**V_{OC} (V)**	**J_{SC} (mA cm^{-2})**	**FF (%)**	**PCE (%)**	**AVT (%)**	**LUE (%)**
D:A=1:2	0.860 (0.859±0.001)	24.7 (24.4±0.2)	68.8 (68.0±0.8)	14.3 (14.2±0.1)	16.1	2.3
D:A=1:3	0.859 (0.857±0.001)	21.3 (21.1±0.1)	63.0 (62.3±0.6)	11.5 (11.3±0.2)	24.2	2.8
D:A=1:5	0.846 (0.845±0.001)	17.4 (17.0±0.3)	62.5 (61.6±0.5)	9.1 (8.9±0.2)	29.1	2.7
D:A=1:8	0.836 (0.835±0.001)	17.0 (16.8±0.2)	56.3 (55.5±0.7)	8.1 (7.9±0.2)	31.3	2.5

[a] The parameters are obtained from 5 independent devices.

Table S13. Photovoltaic parameters of semitransparent devices of D:A=1:3 with different electrode thickness under an illumination of air mass 1.5 global (AM 1.5G), 100 mW cm^{-2}.[a]

Condtion		V_{OC} (V)	J_{SC} (mA cm^{-2})	FF (%)	PCE (%)	AVT (%)	LUE (%)
Ag= 10 nm	Undoped	0.852 (0.851±0.001)	19.9 (19.6±0.2)	59.3 (58.1±0.7)	9.9 (9.6±0.2)	27.1	2.7
	Doped	0.852 (0.850±0.001)	19.8 (19.6±0.2)	65. 7 (64.6±0.7)	10.7 (10.6±0.1)		2.9
Ag= 15 nm	Undoped	0.859 (0.857±0.001)	21.3 (21.1±0.1)	63.0 (62.3±0.6)	11.5 (11.3±0.2)	24.2	2.8
	Doped	0.859 (0.858±0.001)	21.2 (21.1±0.1)	68.2 (67.0±0.6)	12.3 (12.1±0.1)		3.0
Ag= 20 nm	Undoped	0.864 (0.863±0.001)	22.0 (21.9±0.1)	67.9 (67.2±0.6)	13.2 (12.8±0.3)	19.2	2.5
	Doped	0.865 (0.863±0.001)	22.0 (21.5±0.3)	70.4 (69.2±0.9)	13.9 (13.6±0.3)		2.7

[a] The parameters are obtained from 10 independent devices.

Table S14. Detailed parameter on state-of-the-art ST-OSC devices reported in the literature.

Active Layer	PCE (%)	AVT (%)	LUE (%)	Reference
PBFTT: IT-4Cl	9.1	27.6	2.51	[6]
PTB7-Th: IUIC	10.2	31	3.16	[7]
PTB7-Th: ACS8	11.1	28.6	3.17	[8]
PCE10: ICBA:Y8	10.46	26.56	2.78	[9]
PTB7-Th: IEICO-4F	9.06	27.1	2.46	[10]
PFTzTT3TC: ITIC	6.43	26.77	1.72	[11]
PTB7-Th: IEICO-4F	10.83	29.5	3.19	[12]
PM6: Y6: PC71BM	10.2	28.6	2.92	[13]
PTB7-Th: IHIC	8.38	27.9	2.33	[14]
PM6:A-2ThCl:A-4Cl: $PC_{71}BM$	13.02	26.3	3.42	[15]
PM6:BT*P*-eC9	13.1	27.8	3.64	[16]
PM6:Y6-BO:2PACz	11.3	30.0	3.39	[17]
PBDB-TF:Y6:BDC-4 F-C8	11.56	26.58	3.07	[18]

References

[1] F M. J. risch , G. W. Trucks , H. B. Schlegel, G. E. Scuseria, M. A. Robb , J. R. Cheeseman, G. Scalmani, B V. arone, B. Mennucci, G. A. Petersson, Gaussian 16, Revision C.01. Gaussian, Inc., Wallingford CT **2016**.

[2] J. D. Chai & M. Head-Gordon, *Phys. Chem. Chem. Phys.* **2008**, 10, 6615-6620 .

[3] W. Zhao , D. Qian , S. Zhang , S. Li , O. Inganäs , F. Gao and J. Hou, *Adv. Mater.* **2016**, 28, 4734-4739 .

[4] Z. Zheng , J. L. Bredas & V. Coropceanu, *J. Phys. Chem. Lett.* **2016**, 7, 2616-2621.

[5] A. Hexemer, W. Bras, J. Glossinger, E. Schaible, E. Gann, R. Kirian, A. MacDowell, M. Church, B. Rude and H. Padmore, *J. Phys: Confer Ser.* **2010**, *247*, 012007.

[6] W. Su, Q. Fan, X. Guo, , J. Wu, M. Zhang, and Y. Li, *Phys. Chem. Chem. Phys.* **2019**, *21*, 10660-10666.

[7] B. Jia, S. Dai, Z. Ke, C. Yan, W. Ma and X. Zhan, *Chem. Mater.* **2017**, *30*, 239-245.

[8] J. Chen, G. Li, Q. Zhu, X. Guo, Q. Fan, W. Ma and M. Zhang, *J. Mater. Chem. A.* **2019**, *7*, 3745-3751.

[9] C. Zhu, H. Huang, Z. Jia, F. Cai, J. Li, J. Yuan, L. Meng, H. Peng, Z. Zhang, Y. Zou, *Sol. Energy.* **2020**, *204*, 660-666.

[10] Z. Hu, Z. Wang and F. Zhang, *J. Mater. Chem. A.* **2019**, *7*, 7025-7032.

[11] X. Wang, K. Zhu, X. Jing, Q. Wang, F. Li, L. Yu and M. Sun, *ACS Appl. Energy Mater.* **2019**, *3*, 915-922.

[12] R. Xia, C. J. Brabec, H.-L. Yip and Y. Cao, *Joule.* **2019**, *3*, 2241-2254.

[13] B. H. Jiang, H. E. Lee, J. H. Lu, T. H. Tsai, T. S. Shieh, R. J. Jeng and C. P. Chen, *ACS Appl. Mater. Interfaces.* **2020**, *12*, 39496-39504.

[14] J. Zhang, G. Xu, F. Tao, G. Zeng, M. Zhang, Y. M. Yang, Y. Li and Y. Li, *Adv. Mater.* **2019**, *31*, 1807159.

[15] D. Wang, H. Liu, Y. Li, G. Zhou, L. Zhan, H. Zhu, X. Lu, H. Chen and C.-Z. Li, *Joule*. **2021**, *5*, 945-957.

[16] D. Yang, R. Zhang, Y. Shi, X. Guo and M. Zhang, *J. Mater. Chem. C.* **2022**, *10*, 14597-14604.

[17] J. Jing, S. Dong, K. Zhang, Z. Zhou, Q. Xue, Y. Song, Z. Du, M. Ren and F. Huang, *Adv. Energy Mater.* **2022**, *12*, 2200453.

[18] Y. Zhang, D. Luo, C. Shan, Q. Liu, X. Gu, W. Li, W. C. H. Choy and A. K. K. Kyaw, *Sol. RRL.* **2021**, *6*, 2100785.
